# Ten-year trends in opioid prescribing and vaso-occlusive crises in sickle cell disease: a population-based national cohort study (2011–2022)

**DOI:** 10.1016/j.lana.2025.101214

**Published:** 2025-08-21

**Authors:** Kevin Y. Xu, Terri V. Newman, Lakeya S. McGill, Enrico M. Novelli, Cheryl A. Hillery, Joanna L. Buss, Lisa Gong, Ruizhi Huang, Fanghong Dong, Dustin Stwalley, Joanne Salas, Shiyuan A. Liu, Jeffrey F. Scherrer, Tashalee R. Brown, Tae Woo Park, Marc R. LaRochelle, Richard A. Grucza, Charles R. Jonassaint

**Affiliations:** a Department of Psychiatry, Washington University School of Medicine, St. Louis, MO, USA; b Washington University School of Public Health, St. Louis, MO, USA; c Department of Pharmacy and Therapeutics, University of Pittsburgh School of Pharmacy, Pittsburgh, PA, USA; d Division of General Internal Medicine, Department of Medicine, University of Pittsburgh School of Medicine, Pittsburgh, PA, USA; e Division of Classical Hematology, Department of Medicine, University of Pittsburgh School of Medicine, Pittsburgh, PA, USA; f Vascular Medicine Institute, University of Pittsburgh School of Medicine, Pittsburgh, PA, USA; g Division of Hematology-Oncology, Department of Pediatrics, University of Pittsburgh School of Medicine, Pittsburgh, PA, USA; h Institute for Informatics, Data Science and Biostatistics, Washington University School of Medicine, St. Louis, MO, USA; i Department of Family and Community Medicine, Saint Louis University School of Medicine, St. Louis, MO, USA; j The Advanced HEAlth Data (AHEAD) Research Institute, Saint Louis University School of Medicine, St. Louis, MO, USA; k Division of Hematology and Oncology, UCSF School of Medicine, San Francisco, CA, USA; l Department of Psychiatry and Behavioral Neuroscience, Saint Louis University School of Medicine, St. Louis, MO, USA; m Jane and Terry Semel Institute for Neuroscience and Human Behavior at UCLA, Department of Psychiatry and Biobehavioral Sciences, David Geffen School of Medicine at UCLA, Los Angeles, CA, USA; n Department of Psychiatry, University of Pittsburgh School of Medicine, Pittsburgh, PA, USA; o Clinical Addiction Research and Education Unit at Boston University School of Medicine, Boston Medical Center, Boston, MA, USA

**Keywords:** Opioid prescribing, Vaso-occlusive crises, Sickle cell disease, Age disparities, Insurance, Administrative claims, COVID-19

## Abstract

**Background:**

Patterns of opioid prescribing and vaso-occlusive crises (VOCs) are poorly characterized among individuals with sickle cell disease (SCD) across diverse insurance types and age groups. We aimed to evaluate opioid prescribing and VOC trends in publicly and commercially insured individuals with SCD over a 10-year time period in the United States (US).

**Methods:**

We conducted a retrospective cohort study of US administrative claims (2011–2022), analyzing 45,726 commercial and Medicaid beneficiaries with SCD. Primary outcomes were monthly rates of outpatient opioid prescriptions and VOC-related acute care encounters. We used joinpoint regression models to estimate trends without pre-specifying breakpoints, stratified by insurance type (Medicaid vs commercial) and age group (1–12, 13–17, 18–27, 28–45, 46–64 years). Primary outcomes were monthly rates of outpatient opioid prescriptions and VOC-related acute care encounters. We used joinpoint regression models to estimate trends without pre-specifying breakpoints, stratified by insurance type (Medicaid vs commercial) and age group.

**Findings:**

Among 45,726 individuals with SCD (mean age [SD] = 25.1 [16.2]; 39.7% female; 52.9% Medicaid, 47.1% commercial insurance), Medicaid beneficiaries had higher rates than commercial beneficiaries for monthly opioid prescribing (18.3 vs 14.0 per 100) and VOC encounters (16.6 vs 8.2 per 100). Monthly opioid prescribing per 100 people increased with age: 1–12 y = 5.1; 13–17 y = 11.3; 18–27 y = 22.5; 28–45 y = 24.6; 46–64 y = 20.6 per 100. Both Medicaid and commercial beneficiaries experienced declining opioid prescribing beginning in 2011 (commercial monthly percentage change [MPC] = −0.3% [95% CI: −0.3%, −0.2%]; Medicaid MPC = −0.5% [−0.6%, 0.5%]). Down-trending opioid prescribing was not consistently accompanied by up-trending VOCs until the COVID-19 pandemic's onset. Particularly among children and adolescents, VOC-related encounters increased significantly after 2020 across both commercial (MPC = 1.8% [1.5%, 2.2%]) and Medicaid (MPC = 0.6% [0.1%, 1.6%]) beneficiaries.

**Interpretation:**

Opioid prescribing and VOC admissions vary by insurance and age. Opioid prescribing declined from 2011 but was not consistently accompanied by increased VOCs until after COVID-19.

**Funding:**

Analyses of Merative MarketScan Commercial and Multi-State Medicaid Database were funded by grants 10.13039/100000002NIH K12 DA041449 (PI: KYX; data analysts: JLB, DS). Effort for some personnel was supported by P50 MH122351 (KYX, PI: Eric Lenze MD, Michael Avidan MBBCh), K08 K08 DA061258 (KYX), the 10.13039/100005386American Psychiatric Association (APA) Psychiatric Research Fellowship (with funding by 10.13039/100000026NIDA and the 10.13039/100005386APA, KYX), 10.13039/100000002NIH K12NS130673 (LSM), 10.13039/100000002NIH L60HL170453 (LSM), and the St. Louis University Research Institute Fellowship (RAG, JS, JFS, RH); these grants did not fund Merative MarketScan Commercial and Multi-State Medicaid Database data pull.

**Research in context**

**Evidence before this study**

To identify prior research investigating opioid prescribing patterns among people with SCD, we searched PubMed from inception to November 16, 2023, using the search terms “(Sickle Cell Disease” [All Fields] OR “Sickle Cell Anemia” [All Fields]) AND (“Opioid” [All Fields] or “Opiate” [All Fields]) AND (“Vaso-occlusive” [All Fields] or “Hospitalization” [All Fields] or “Admission” [All Fields]). We found that opioid prescribing patterns and related health care utilization for people with sickle cell disease (SCD) are poorly characterized, especially across diverse age groups and insurance types. Prior studies have been limited by short time frames and a focus on commercially insured populations, an important limitation given that most individuals living with SCD are publicly insured. To address this gap in research, we comprehensively evaluated patterns of opioid prescribing and VOC admissions in both publicly and commercially insured individuals with SCD over a 10+ year time period in the U.S.

**Added value of this study**

Using the Merative™ MarketScan® Multi-State Commercial and Medicaid Databases, the present study is one of the only analyses to comprehensively evaluate opioid prescribing patterns and healthcare utilization across both public and private payers for individuals with SCD, using national-level data spanning over a decade. We estimated trends in monthly opioid prescribing and VOC admission rates via joinpoint regressions, which can operate without prior specification of where break points may occur and are therefore useful for hypothesis generation and examination of emerging evidence.

Medicaid beneficiaries experienced higher monthly rates of opioid prescribing and VOC admissions compared to the commercial cohort. Adults (>18 years old) generally had higher opioid prescribing and VOC admission rates than children and adolescents across both insurance cohorts. From 2011 to 2022, both Medicaid and commercial enrollees experienced an overall downtrend in monthly opioid prescribing that began prior to the 2016 CDC opioid prescribing policies. Particularly in the post-COVID-19 years, we observed significant increases in VOC admissions for both Medicaid and commercial enrollees, which were most concentrated in the pediatric and young adult population. Contrary to our expectations, the downtrend in opioid prescribing was not accompanied by consistent increases in VOCs until the onset of the COVID-19 pandemic.

**Implications of all the available evidence**

Our data provide new insights into disparities in opioid prescribing and VOC admissions by insurance status and age. Specifically, we observed that publicly insured individuals, particularly those on Medicaid, face a higher burden of both opioid prescribing and VOC admissions compared to their commercially insured counterparts. While children and adolescents typically experience lower rates of opioid prescribing and VOCs than adults, this study reveals a potential rise in pediatric VOC admissions since 2020, across both insurance types. Importantly, our analysis did not rely on predefined time points to detect changes. Instead, we identified shifts in trends directly from the data, allowing for a more flexible and comprehensive understanding of how opioid prescribing and VOC utilization have evolved. Notably, we did not observe a straightforward correlation between declining opioid prescribing rates and increasing VOC admissions, suggesting that more complex and nuanced factors are driving the rise in VOC-related acute care utilization across different age groups and payer types.

## Introduction

While the treatment of sickle cell disease (SCD) has seen breakthroughs in the development of disease-modifying and curative therapies, vaso-occlusive crises (VOCs) continue to be a leading cause of hospital admissions in individuals with SCD. As outlined in recent American Society of Hematology guidelines, opioid medications play an important role in the acute treatment of VOCs and chronic management of pain.^1^ Despite the burden of pain associated with SCD and the well-established role of opioids as an effective pain relief strategy,^1^ there is concern that individuals with SCD often experience obstacles to accessing appropriate opioid treatments.^2^ Previous literature has documented a myriad of barriers to receiving opioid medications among individuals with SCD, including stigmatization by clinicians^3^ and an increasing number of policies intended to reduce opioid prescribing, such as the 2016 US Centers for Disease Control and Prevention (CDC) prescribing guidelines.^4^

Although prior studies have raised concerns that these policies may contribute to decreased opioid prescribing and increased acute care utilization,^4^ many have relied on study designs that assume a predefined inflexion point, typically the 2016 CDC guidelines, as a primary driver of change. While valuable in the context of evaluating general population responses to policy change, this approach may not fully account for the broader and more complex set of factors that influence clinical practice and patient outcomes over time. For example, trends in SCD-related outcomes across the 2011–2022 period could potentially reflect a confluence of shifting policies, care models, structural barriers, and the disruptive impact of the COVID-19 pandemic. To better capture this complexity, we used a flexible, data-driven approach to identify shifts in trends without relying on user-defined change points. This allows for the possibility that major changes in practice may have occurred outside of, or in addition to, the 2016 guidelines.

An additional limitation is that many national-level studies have focused primarily on commercially insured populations, limiting their relevance to the large proportion of individuals with SCD who are publicly insured.^5^, ^6^, ^7^ This is an important gap in the literature, as multiple studies have highlighted worse outcomes in publicly insured individuals with SCD compared to their commercially insured peers.^5^^,^^8^ Other studies have raised concern that SCD-related acute care utilization disproportionately impacts children and young adults, populations more likely to be covered by Medicaid.^6^^,^^7^ Despite these concerns, comprehensive data evaluating how age and insurance type influence long-term trajectories of opioid prescribing and VOC-related outcomes, especially in the post-COVID-19 era, remain limited.

Given these gaps, the goal of this study was to assess ten-year trends in opioid prescription fills and VOC-related acute care encounters among individuals with SCD using a large, geographically diverse administrative claims database in the US from 2011 to 2022. By stratifying our analyses by insurance type (Medicaid vs commercial insurance) and age group (1–12, 13–17, 18–27, 28–45, and 46–64 years), we aimed to provide a comprehensive understanding of evolving healthcare utilization patterns in a population that has been historically underserved.

## Methods

### Study overview

In this retrospective cohort study, we analyzed the combined Merative™ MarketScan® Commercial and Multi-State Medicaid Databases (2011–2022) to estimate joinpoint regression models. The MarketScan database includes linked inpatient, ambulatory and office-based, and outpatient pharmacy claims for >200 million children and adults (ages 1–64 years) in the US, allowing us to evaluate over ten years of claims data.

### Justification for joinpoint regression

There are many factors that may influence SCD-related outcomes across the 2011–2022 period. To our knowledge, no national-level study has evaluated trends in opioid prescribing and VOC-related encounters across a ten-year period spanning major events such as the 2016 CDC opioid prescribing guidelines, the COVID-19 public health emergency, and the 2022 revisions to the CDC guidelines. Given the potential for multiple, overlapping influences on clinical practice during this period, we thus applied joinpoint models (segmented linear regression) to model long-term trend data *without prespecifying* where change points would occur.^9^^,^^10^ This data-driven approach differs from traditional interrupted time series-based methods, which require a priori specification of change points, and thus may miss important inflexion points or overemphasize expected ones.

### Observation window and dataset

The observation window for the study spanned from January 1, 2011 to December 31, 2022. Data cleaning, linkage of claims, and de-identification were conducted by Merative. Each filled outpatient prescription contained data on generic name, product name, continuous days' supply, quantity prescribed, and dosage (converted into morphine-milligram equivalents, refer to Supplementary Data, eMethods 1). The MarketScan data are fully compliant with the Health Insurance Portability and Accountability Act (1996). All data were de-identified, and analyses were determined to be exempt research by the Washington University Human Research Protection Office. Due to heterogeneity in the socioeconomic characteristics of Medicaid and commercial (private, employer-based) insurance beneficiaries, we examined potential disparities in opioid prescribing and VOC-related encounters between Medicaid beneficiaries with SCD and commercially insured peers, analyzing the Medicaid and commercial cohorts separately.

### Study population

We evaluated a cohort of children and adults with SCD, all of whom had prescription medication coverage. Using established International Classification of Diseases 9th (ICD-9) and 10th revision (ICD-10) codes, we defined the diagnosis of SCD by requiring at least one inpatient or two outpatient encounters with an SCD diagnosis at least thirty days apart at any point during insurance enrollment (Supplementary Material, eMethods 1).^11^, ^12^, ^13^ Similar to past analyses of aggregate population-based outcomes at the month level,^4^ we did not require continuous insurance enrollment.

### Outcomes and covariates

The primary outcome variables were monthly rates of filled outpatient opioid prescriptions and monthly rates of VOC-related acute care encounters. Because absolute counts for opioid prescriptions and VOC-related encounters may be susceptible to time-varying exposure at the population level,^10^ the dependent variables were computed as percentages per 100 people and their estimated standard errors. The definition of opioids (which excludes buprenorphine) is included in Supplementary Material, eMethods 1.

We computed monthly opioid prescription rates by dividing the total number of filled opioid prescriptions per month by the number of enrolled individuals in the month. Primary outcome variables were stratified by age group and insurance type. We classified each outpatient opioid prescription by date and month of fill. Because opioid prescribing for VOCs is often intermittent, no restrictions were placed on refill gaps (i.e., maximum number of days allowed between the end of one prescription and the start date of the next). VOC-related acute care encounters were identified using inpatient insurance claims with diagnoses for SCD with crisis (Supplementary Material, eMethods 1) and estimated as a monthly rate by dividing the total number of monthly events by the number of individuals enrolled in each month. We limited claims to encounters in acute care settings (emergency room admissions, urgent medical care clinics visits, inpatient hospitals, ambulances), thus mitigating misclassification bias that would occur from the inclusion of outpatient codes. While claims for VOC admissions are highly specific (>95%), they are less sensitive and may undercount VOC-related encounters^13^^,^^14^; we thus evaluated all-cause acute care encounters^15^ as a secondary outcome (refer to definition in Supplementary Material, eMethods 1).

Covariates included age (years), sex (as recorded in insurance claims), race and ethnicity (data only available for Medicaid beneficiaries), and region of the US. Secondary analyses were conducted analyzing monthly rates of filled opioids, stratified by the specific daily dose (converted to morphine milligram equivalents) and days' supply of each fill. To calculate continuous days' supply, in the event that individuals had multiple overlapping fills, we computed the sum of days supplied.

### Statistical analysis

We first computed descriptive statistics for baseline covariates. Continuous variables were expressed as means, and categorical variables were expressed by counts and percentages (%). To mitigate left truncation bias, we took into account differences in the distribution structure of age (at the time of filled opioid prescription or acute care encounter) across years. Age-specific monthly rates were calculated using the number of events in each age group per month divided by the number of individuals in each age group per month, with rates reported per 100 individuals. The age-standardized monthly rates are calculated by multiplying the monthly age-specific rates by the corresponding weights (Supplementary Material, eMethods 1). We also took into account differences in insurance types and computed insurance-specific estimates by public (Medicaid) and private (commercial data) insurance separately.

To analyze temporal changes in monthly opioid prescriptions and acute care encounters, joinpoint regression was performed. In our joinpoint models, the monthly percentage change (MPC) between trend-change points and the average monthly percentage change (AMPC) over the entire study period were calculated.^9^ Empirical quantiles were used for confidence intervals (CI) calculation. To account for heteroscedasticity (non-constant variance across data points), we used weighted least squares estimation models. To account for potential skew, we employed a log-transformation of outcome variables to fit a log-linear joinpoint regression, which models proportional changes over time while accounting for the non-normal distribution of the data. Our regression coefficients were estimated by weighted least squares, with the weight of each data point estimated as the square of the response divided by the variance at each data point.

We identified all possible combinations of joinpoints and selected the best-fitting model based on the weighted Bayesian Information Criterion (BIC) method.^9^ We allowed as few as 4 observed time points in the middle of a line segment and 3 observed time points in the beginning and end of a line segment, the fewest points necessary to test whether the last observed time point is consistent with prior linear trends. We permitted as few as 0 join points (the null hypothesis). While it is possible to specify a maximum of two fewer than the total number of observed time points, we specified a maximum of 5 joinpoints,^9^ to avoid multiple testing and overfitting. We used the grid search method to restrict join point locations to occur only on observed monthly time points.

Statistically significant changes in trends, described by the MPC, and 95% CIs were reported. Analyses were stratified by age at time of opioid prescription or event (1–12 years [childhood], 13–17 years [early to mid-adolescence], 18–27 years [late adolescence/transition age/emerging adulthood], 28–45 years [early to mid-adulthood], and 46–64 years [late adulthood]). We conducted analyses adjusting for seasonal variations to evaluate the robustness of our observed temporal trends, which apply seasonal-trend decomposition based on Loess smoothening.^16^

Data were analyzed separately using SAS 9.4 and Joinpoint Regression Program, Version 5.1.0.0 (Statistical Research and Applications Branch, National Cancer Institute). All hypothesis testing was two-tailed, with statistical significance set at two-sided p < 0.05. Data analyses were performed from November 20, 2023 through April 30, 2025. The STROBE (Strengthening the Reporting of Observational Studies in Epidemiology) and RECORD-PE guidelines for the reporting of studies conducted using observational routinely collected health data statement for pharmacoepidemiology were followed.

### Role of the funding source

The funding sources had no role in study design, data collection, data analysis, data interpretation, writing of the report, or the decision to submit the paper for publication.

## Results

### Summary of clinical characteristics

As illustrated in Table 1, our sample consisted of 45,726 individuals diagnosed with SCD. The mean age was 25.1 years (SD = 16.2), and 39.7% (n = 18,136) were male. Overall beneficiary status was 52.9% Medicaid (n = 24,180) and 47.1% commercial insurance (n = 21,546). 61.7% of the Medicaid cohort (n = 14,925) and 58.8% of the commercial cohort (n = 12,665) were identified as female. 84.5% of the Medicaid cohort (n = 18,266) was identified as non-Hispanic Black (race and ethnicity data unavailable for commercial claims). 63.1% of the commercially insured cohort (n = 13,598) and 55.5% of the Medicaid cohort (n = 13,425) had at least one outpatient opioid prescription; 49.9% (n = 10,742) and 53.4% (n = 12,913) had at least one VOC-related acute care encounter respectively.

**Table 1:** Characteristics of the analytic sample.

	All enrollees	Insurance status		Age at time of enrollment				
		Commercial	Medicaid	1–12 years	13–17 years	18–27 years	28–45 years	45–64 years
	n = 45,726	n = 21,546	n = 24,180	n = 12,037	n = 4574	n = 10,378	n = 12,595	n = 6142
Sex^a^								
Male (%)	18,136 (39.7)	8881 (41.2)	9255 (38.3)	6204 (51.5)	2220 (48.5)	3515 (33.9)	3961 (31.5)	2236 (36.4)
Female (%)	27,590 (60.3)	12,665 (58.8)	14,925 (61.7)	5833 (48.5)	2354 (51.5)	6863 (66.1)	8634 (68.6)	3906 (63.6)
Insurance status								
Medicaid (%)	24,180 (52.9)	–	24,180 (100)	7868 (32.5)	2672 (11.1)	6070 (25.1)	5570 (23.0)	2000 (8.3)
Commercial (%)	21,546 (47.1)	21,546 (100)	–	4169 (19.4)	1902 (8.8)	4308 (20.0)	7025 (32.6)	4142 (19.2)
Age at time of enrollment								
Mean age (SD)	25.1 years (16.2)	29.1 years (16.5)	21.5 years (15.0)	–	–	–	–	–
1–12 years, (%)	1203 (100)	4169 (19.4)	7868 (32.5)	1203 (100)	–	–	–	–
13–17 years (%)	4574 (100)	1902 (8.8)	2672 (11.1)	–	4574 (100)	–	–	–
18–27 years (%)	10,378 (100)	4308 (20.0)	6070 (25.1)	–	–	10,378 (100)	–	–
28–45 years (%)	12,595 (100)	7025 (32.6)	5570 (23.0)	–	–	–	12,595 (100)	–
45–64 years (%)	6142 (100)	4142 (19.2)	2000 (8.3)	–	–	–	–	6142 (100)
Race and ethnicity^b^								
Non-Hispanic White (%)	924 (4.3)	–	924 (4.3)	256 (3.3)	83 (3.1)	208 (3.4)	189 (3.4)	188 (9.4)
Non-Hispanic Black (%)	18,266 (84.8)	–	18,266 (84.8)	5573 (70.8)	1857 (69.5)	4724 (77.8)	4640 (83.3)	1472 (73.6)
Hispanic (%)	336 (1.6)	–	336 (1.6)	118 (1.5)	34 (1.3)	73 (1.2)	91 (1.6)	20 (1.0)
Other (%)	3256 (15.1)	–	3256 (15.1)	1461 (18.6)	575 (21.5)	718 (11.8)	328 (5.9)	174 (8.7)
Unknown (%)	1398 (6.5)	–	1398 (6.5)	460 (5.9)	123 (4.6)	347 (5.7)	322 (5.8)	146 (7.3)
Region of the United States^c^								
Northeast (%)	3675 (17.1)	3675 (17.1)	–	675 (16.2)	294 (15.5)	669 (15.5)	1211 (17.2)	826 (19.9)
North Central (%)	3177 (14.8)	3177 (14.8)	–	665 (16.0)	316 (16.6)	678 (15.7)	940 (13.4)	578 (14.0)
South (%)	12,872 (59.7)	12,872 (59.7)	–	2530 (60.7)	1123 (59.0)	2617 (60.8)	4283 (61.0)	2319 (56.0)
West (%)	1499 (6.9)	1499 (6.9)	–	242 (5.8)	127 (6.7)	280 (6.5)	508 (7.2)	342 (8.3)
Unknown (%)	323 (1.5)	323 (1.5)	–	57 (1.4)	42 (2.2)	64 (1.5)	83 (1.2)	77 (1.9)
Outpatient opioid prescriptions								
≥1 outpatient opioid prescription (%)	27,023 (59.1)	13,598 (63.1)	13,425 (55.5)	5472 (45.5)	2895 (63.3)	6425 (61.9)	8054 (64.0)	4177 (68.0)
Mean number of prescriptions per month per 100 people^d^ (SD)	16.8 (11.2)	14.0 (2.1)	18.3 (5.8)	5.1 (1.3)	11.3 (2.8)	22.5 (10.1)	24.6 (12.4)	20.6 (9.2)
≥1 prescription with 90 MMEs (%)	6340 (13.9)	2887 (13.4)	3453 (14.3)	405 (3.4)	717 (15.7)	1976 (19.0)	2219 (17.6)	1023 (16.7)
≥1 prescription with <7 day supply (%)	21,502 (47.0)	10,335 (48.0)	11,167 (46.2)	4910 (40.8)	2640 (57.7)	5216 (50.3)	5842 (46.4)	2894 (47.1)
≥1 prescription with 7–14 day supply (%)	14,016 (30.7)	6519 (30.3)	7497 (31.0)	3069 (25.5)	1613 (35.3)	3361 (32.4)	3828 (30.4)	2145 (34.9)
≥1 prescription with 15–28 day supply (%)	7900 (17.3)	3616 (16.8)	4284 (17.7)	1012 (8.4)	740 (16.2)	2120 (20.4)	2510 (19.9)	1518 (24.7)
≥1 prescription with >28 day supply (%)	8485 (18.6)	3754 (17.4)	4731 (19.6)	638 (5.3)	654 (14.3)	2189 (21.1)	3132 (24.9)	1872 (30.5)
Acute care encounters								
≥1 VOC-related acute care encounter (%)	23,655 (51.7)	10,742 (49.9)	12,913 (53.4)	7401 (61.5)	2933 (64.1)	5607 (54.0)	5634 (44.7)	2077 (33.8)
Mean number of monthly VOC-related acute care encounters per 100 people^e^ (SD)	12.5 (9.3)	8.2 (1.0)	16.6 (1.5)	6.2 (1.7)	9.5 (2.8)	21.3 (9.2)	18.7 (11.4)	7.1 (3.6)
≥1 all-cause acute care encounter (%)	42,090 (92.1)	19,361 (89.9)	22,729 (94.0)	11,243 (93.4)	4283 (93.6)	9617 (92.7)	11,402 (90.5)	5545 (90.3)
Mean number of monthly all-cause acute care encounters per 100 people^f^ (SD)	37.9 (13.1)	28.6 (2.9)	44.5 (4.2)	31.0 (5.6)	34.8 (5.9)	44.9 (13.2)	43.1 (17.9)	35.6 (12.7)

MME = morphine milligram equivalents; SD = standard deviation; VOC = vaso-occlusive crisis.

a Sex, as reported in insurance claims.

b Race and ethnicity data was only available for Medicaid enrollees.

c Region of the US was only available for Commercial enrollees.

d Among those with >1 outpatient opioid prescription.

e Among those with >1 VOC-related acute care encounter.

f Among those with >1 all-cause acute care encounter.

Table 1 shows that adults had higher overall mean monthly opioid prescribing rates than children and adolescents (1–12 years = 5.1, 13–17 years = 11.3, 18–27 years = 22.5, 28–45 years = 24.6, 46–64 years = 20.6 per 100 individuals). Mean monthly rates of VOC-related acute care encounters were the highest in emerging (transition-age) adults who were 18–27 years of age: 1–12 years = 6.2, 13–17 years = 9.5, 18–27 years = 21.3, 28–45 years = 18.7, 46–64 years = 7.1 per 100 individuals.

Compared to commercially insured individuals, the Medicaid cohort was younger (mean age 21.5 years versus 29.1 years, p < 0.001). Medicaid beneficiaries, compared to commercially insured peers, also had a higher mean monthly rate of opioid prescribing (18.3 vs 14.0 per 100 individuals, p < 0.001) and had a higher mean monthly rate of VOC-related encounters (16.6 vs 8.2 per 100 individuals, p < 0.001) and all-cause acute care encounters (44.5 vs 28.6 per 100 individuals, p < 0.001).

### Overall opioid prescribing trends

**Fig. 1:**
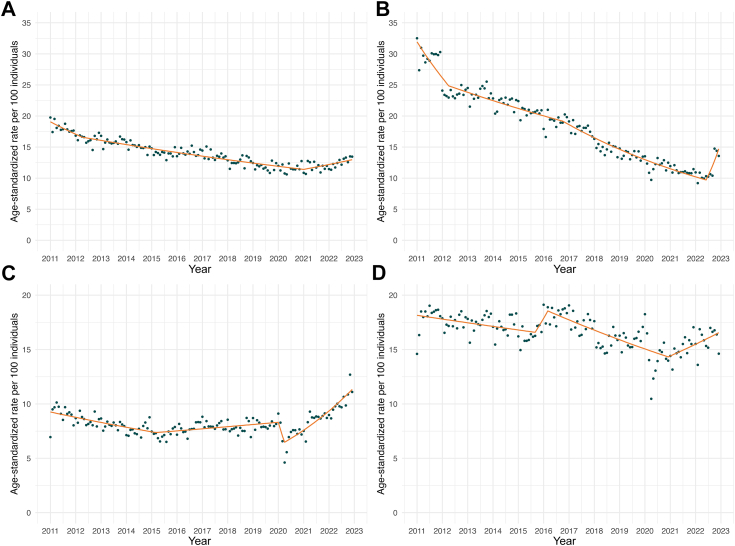
**Joinpoint (JP) models illustrating age-standardized rates of monthly outpatient opioid prescribing and VOC-related acute care encounters, 2011–2022 stratified by insurance.** Joinpoint regression models identified significant trend changes marked by breakpoints for monthly age-standardized outpatient opioid prescribing and VOC-related acute care encounters by insurance type from 2011 to 2022. **(a) Monthly age-standardized opioid prescribing per 100 individuals among commercial enrollees.** Trend periods are as follows: January 2011–April 2012, MPC = −0.92% (95% CI: −2.91, −0.45)∗; April 2012–January 2021, MPC = −0.36% (−0.39, −0.30)∗; January 2021–December 2022, MPC = 0.56% (0.18, 1.25)∗. **(b) Monthly age-standardized opioid prescribing per 100 individuals among Medicaid enrollees.** Trend periods are as follows: January 2011–April 2012, MPC = −1.66% (95% CI: −2.93, −1.08)∗; April 2012–October 2016, MPC = −0.48% (−0.57, −0.29)∗; October 2016–June 2022, MPC = −1.00% (−1.13, −0.91)∗; June 2022–December 2022, MPC = 7.17% (3.11, 15.40)∗. **(c) Monthly age-standardized VOC-related acute care encounters per 100 individuals among commercial enrollees.** Trend periods are as follows: January 2011–March 2015, MPC = −0.46% (95% CI: −0.64, −0.33)∗; March 2015–January 2020, MPC = 0.21% (0.10, 0.41)∗; January 2020–April 2020, MPC = −7.96% (−9.88, −1.15)∗; April 2020–December 2022, MPC = 1.76% (1.48, 2.15)∗. **(d) Monthly age-standardized VOC-related acute care encounters per 100 individuals among Medicaid enrollees.** Trend periods are as follows: January 2011–September 2015, MPC = −0.16% (95% CI: −0.38, −0.04)∗; September 2015–March 2016, MPC = 1.88% (0.02, 5.03)∗; March 2016–December 2020, MPC = −0.45% (−0.69, −0.35)∗; December 2020–December 2022, MPC = 0.60% (0.11, 1.58)∗. MPC = monthly percentage change (95% CI); VOC = vaso-occlusive crisis. ∗Indicates MPC values significantly different from zero at α = 0.05 level.

**Fig. 2:**
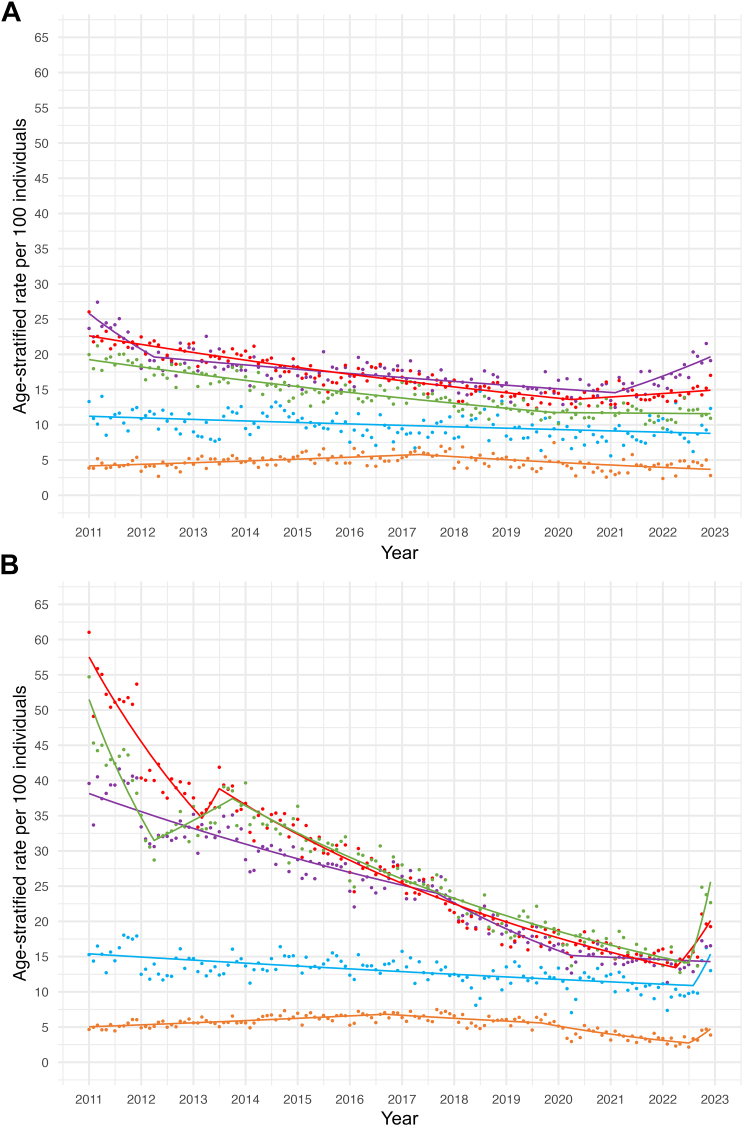
**Joinpoint (JP) models illustrating age- and insurance-stratified rates of monthly outpatient opioid prescribing, 2011–2022.** Joinpoint regression models identified trend changes in monthly age-stratified outpatient opioid prescribing by age group and insurance type from 2011 to 2022. Different colored lines represent distinct age groups (1–12, 13–17, 18–27, 28–45, 46–64 years). **(a) Monthly age-stratified opioid prescribing per 100 individuals among commercial enrollees across age groups.** Trend periods are as follows. **For children, 1–12 years of age (orange line):** January 2011–May 2017, MPC = 0.43% (95% CI: 0.29, 0.62)∗; May 2017–December 2022, MPC = −0.68% (−1.01, −0.46)∗. **For adolescents, 13–17 years of age (blue line):** January 2011–December 2022, MPC = −0.17% (−0.25, −0.11)∗. **For adults, 18–27 years of age (purple line):** January 2011–April 2012, MPC = −1.81% (−3.32, −1.09)∗; April 2012–February 2021, MPC = −0.28% (−0.34, −0.23)∗; February 2021–December 2022, MPC = 1.37% (0.77, 2.36)∗. **For adults, 28–45 years of age (red line):** January 2011–April 2020, MPC = −0.46% (−0.50, −0.42)∗; April 2020–December 2022, MPC = 0.29% (0.01, 0.77)∗. **For adults, 46–64 years of age (green line):** January 2011–December 2019, MPC = −0.46% (−0.55, −0.42)∗; December 2019–December 2022, MPC = −0.04% (−0.32, 1.11). **(b) Monthly age-stratified opioid prescribing per 100 individuals among Medicaid enrollees across age groups.** Trend periods are as follows. **For children, 1–12 years of age (orange line):** January 2011–September 2016, MPC = 0.45% (95% CI: 0.34, 0.61)∗; September 2016–September 2019, MPC = −0.55% (−1.15, −0.01)∗; September 2019–July 2022, MPC = −2.08% (−10.46, −1.51)∗; July 2022–December 2022, MPC = 11.65% (−0.38, 37.55). **For adolescents, 13–17 years of age (blue line):** January 2011–August 2022, MPC = −0.25% (−0.33, −0.20)∗; August 2022–December 2022, MPC = 8.99% (−0.17, 29.02). **For adults, 18–27 years of age (purple line):** January 2011–September 2017, MPC = −0.58% (−0.64, −0.51)∗; September 2017–April 2020, MPC = −1.47% (−6.19, −1.15)∗; April 2020–December 2022, MPC = −0.18% (−0.66, 0.80). **For adults, 28–45 years of age (red line):** January 2011–March 2013, MPC = −1.93% (−2.31, −1.69)∗; March 2013–July 2013, MPC = 2.91% (−0.36, 5.69); July 2013–April 2022, MPC = −1.01% (−1.07, −0.97)∗; April 2022–December 2022, MPC = 5.18% (2.52, 10.23)∗. **For adults, 46–64 years of age (green line):** January 2011–April 2012, MPC = −3.23% (−4.15, −2.54)∗; April 2012–October 2013, MPC = 0.97% (0.43, 2.12)∗; October 2013–July 2022, MPC = −0.93% (−0.99, −0.89)∗; July 2022–December 2022, MPC = 12.74% (6.98, 22.27)∗. MPC = monthly percentage change (95% CI); ∗Indicates MPC values significantly different from zero at α = 0.05 level.

Fig. 1 shows the joinpoint regression models for age-standardized monthly opioid prescribing, and Fig. 2 shows the age-stratified monthly opioid prescribing rates for both commercial and Medicaid beneficiaries. Full models are depicted in Supplementary Material, eMethods 2–6.

Among commercial beneficiaries, overall monthly opioid prescribing decreased from 19.1 per 100 individuals in January 2011 to 13.5 per 100 individuals in December 2022, evidenced by an average monthly percentage change (MPC) of −0.3% (Supplementary Material, eMethods 2), with monthly opioid prescribing declining among all age groups (Fig. 2, Supplementary Material, eMethods 5).

Among Medicaid beneficiaries, monthly opioid prescribing also declined overall from January 2011 (32.5 per 100 individuals) to December 2022 (13.6 per 100 individuals), with an average MPC of −0.5% (Fig. 1, Supplementary Material, eMethods 2). There were age differences in opioid prescribing such that monthly opioid prescribing declined in adults (18 years and older) but not children and adolescents (Fig. 2, Supplementary Material, eMethods 5).

We conducted secondary analyses that applied seasonal-trend decomposition to the BIC method (Supplementary Material, eMethods 4); overall opioid prescribing trends did not differ from parent models.

### Overall trends in VOC-related encounters

The monthly rates of age-standardized VOC-related acute care encounters are shown in Fig. 1. From 2011 to 2022, the commercial cohort experienced a monthly average percent change increase of 0.1% in VOC-related encounters (Supplementary Material, eMethods 2), rising from 7.0 per 100 individuals in January 2011 to 11.1 per 100 individuals in December 2022. For age-stratified analyses, all age groups saw significantly increasing rates of monthly VOC-related encounters from 2011 to 2022, aside from the 18-27 year-old cohort, which experienced no mean increase in monthly rates of VOC-related encounters (Fig. 2, Supplementary Material, eMethods 5).

In contrast, age-standardized VOC-related encounters in the Medicaid cohort showed an overall decrease in monthly rates over the study period (MPC = −0.1%, Fig. 1, Supplementary Material, eMethods 2). Age-stratified analyses showed heterogeneity in VOC-related encounter trajectories by age: younger age groups generally experienced increases in VOC-related acute care encounters; older age groups, despite having the highest monthly rates of VOCs, saw significant decreases in VOC-related acute care encounters over time (Fig. 2, Supplementary Material, eMethods 5).

In secondary analyses that applied seasonal-trend decomposition to the BIC method (Supplementary Material, eMethods 4), monthly VOC-related encounter trends did not differ from parent models.

### Temporal changes in opioid prescribing

Among commercially insured individuals, we observed a reduction in monthly age-standardized opioid prescribing in two time segments: from January 2011 to April 2012 (MPC = −0.9%) and from April 2012 to January 2021 (MPC = −0.4%, Fig. 1, Supplementary Material, eMethods 2). In the year prior to the start of the COVID-19 pandemic, each of the five age groups experienced a decline in opioid prescribing. After 2020, pediatric populations saw decreases in opioid prescribing through the end of the observation period, whereas adults saw either no change in prescribing (46–64-year-old group) or increases in prescribing (18–27-year-olds; 28–45-year-olds) (Fig. 2, Supplementary Material, eMethods 5). Among Medicaid beneficiaries, we also observed an overall reduction in age-standardized prescribing across three time periods: from January 2011 to April 2012 (MPC = −1.7% [95% CI: −2.9%, −1.1%]), from April 2012 to October 2016 (MPC = −0.5% [−0.6%, −0.3%]), and an accentuation in downtrending opioid prescriptions from October 2016 to June 2022 (MPC = −1.0% [95% CI: −1.1%, −0.9%]) (Fig. 2, Supplementary Material, eMethods 5). Whereas all five age groups in the Medicaid cohort experienced down-trending opioid prescriptions in the year prior to the start of the pandemic, there were age disparities in post-COVID-19 opioid prescribing trajectories, such that adults (28–45 year old and 46–64 year old cohorts) experienced increases in opioid prescribing in 2021 that were not seen among children, adolescents, and transitional-age adults (Supplementary Material, eMethods 5).

### Temporal changes in VOC-related encounters

**Fig. 3:**
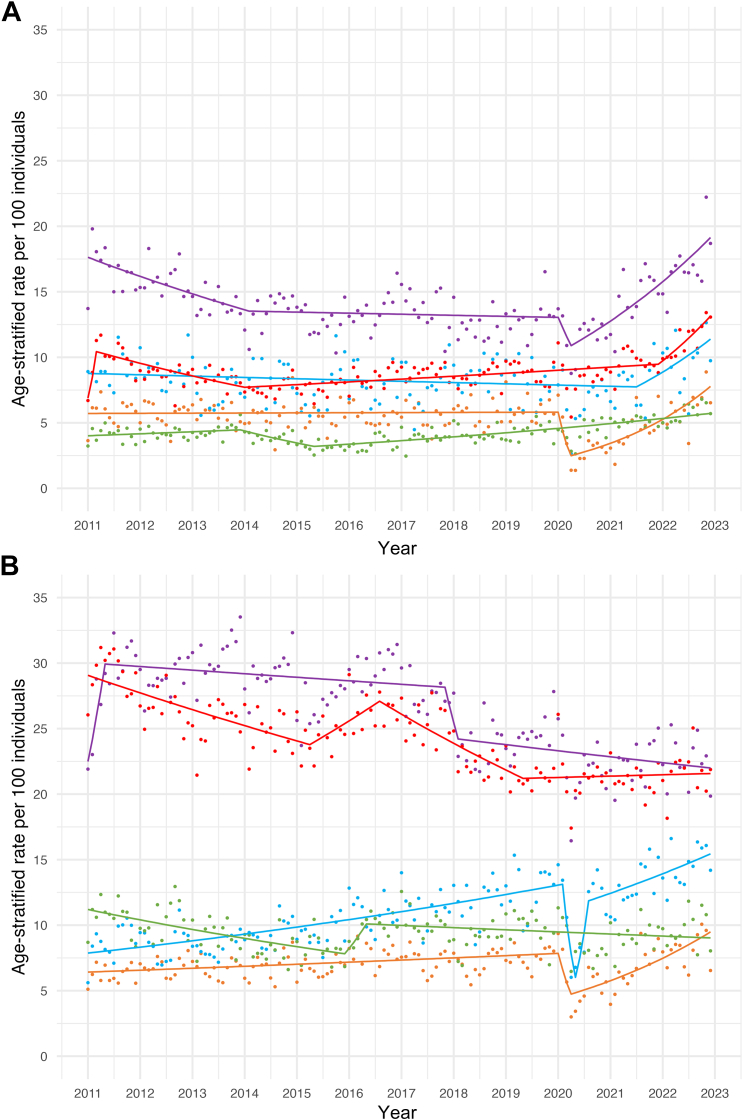
**Joinpoint (JP) models illustrating age- and insurance-stratified rates of VOC-related acute care encounters, 2011–2022.** Joinpoint regression models identified trend changes in monthly age-stratified VOC-related acute care encounters by age group and insurance type from 2011 to 2022. Different colored lines represent distinct age groups (1–12, 13–17, 18–27, 28–45, 46–64 years). **(a) Monthly age-stratified VOC-related acute care encounters per 100 individuals among commercial enrollees across age groups.** Trend periods are as follows. **For children, 1–12 years of age (orange line):** January 2011–January 2020, MPC = 0.02% (95% CI: −0.08, 0.11); January 2020–April 2020, MPC = −24.58% (−29.23, −5.48)∗; April 2020–December 2022, MPC = 3.62% (2.72, 4.92)∗. **For adolescents, 13–17 years of age (blue line):** January 2011–July 2021, MPC = −0.10% (−0.26, −0.02)∗; July 2021–December 2022, MPC = 2.30% (0.24, 13.84)∗. **For adults, 18–27 years of age (purple line):** January 2011–February 2014, MPC = −0.72% (−1.49, 0.74); February 2014–January 2020, MPC = −0.05% (−3.47, 4.17); January 2020–April 2020, MPC = −5.84% (−8.46, 3.97); April 2020–December 2022, MPC = 1.78% (0.40, 2.67)∗. **For adults, 28–45 years of age (red line):** January 2011–March 2011, MPC = 23.06% (−0.48, 39.16); March 2011–January 2014, MPC = −0.89% (−1.65, 0.15); January 2014–December 2021, MPC = 0.22% (0.10, 0.31)∗; December 2021–December 2022, MPC = 2.77% (1.15, 7.39)∗. **For adults, 46–64 years of age (green line):** January 2011–December 2013, MPC = 0.31% (−0.10, 1.02); December 2013–May 2015, MPC = −1.94% (−10.88, −0.79)∗; May 2015–December 2022, MPC = 0.64% (0.53, 0.76)∗. **(b) Monthly age-stratified VOC-related acute care encounters per 100 individuals among Medicaid enrollees across age groups.** Trend periods are as follows. **For children, 1–12 years of age (orange line):** January 2011–January 2020, MPC = 0.19% (95% CI: 0.11, 0.27)∗; January 2020–April 2020, MPC = −15.48% (−18.94, −2.71)∗; April 2020–December 2022, MPC = 2.19% (1.56, 3.13)∗. **For adolescents, 13–17 years of age (blue line):** January 2011–February 2020, MPC = 0.47% (0.40, 0.56)∗; February 2020–May 2020, MPC = −22.87% (−28.45, −10.43)∗; May 2020–August 2020, MPC = 25.38% (8.58, 35.97)∗; August 2020–December 2022, MPC = 0.95% (0.30, 1.45)∗. **For adults, 18–27 years of age (purple line):** January 2011–May 2011, MPC = 7.41% (0.48, 21.44)∗; May 2011–November 2017, MPC = −0.08% (−0.18, 0.004); November 2017–February 2018, MPC = −4.91% (−6.25, −0.45)∗; February 2018–December 2022, MPC = −0.17% (−0.30, 0.07). **For adults, 28–45 years of age (red line):** January 2011–April 2015, MPC = −0.39% (−0.56, −0.27)∗; April 2015–August 2016, MPC = 0.82% (0.23, 5.36)∗; August 2016–May 2019, MPC = −0.74% (−1.64, −0.53)∗; May 2019–December 2022, MPC = 0.04% (−0.12, 0.28). **For adults, 46–64 years of age (green line):** January 2011–December 2015, MPC = −0.61% (−0.90, −0.41)∗; December 2015–May 2016, MPC = 5.25% (0.72, 11.57)∗; May 2016–December 2022, MPC = −0.14% (−0.30, −0.04)∗. VOC = vaso-occlusive crisis; MPC = monthly percentage change (95% CI). ∗Indicates MPC values significantly different from zero at α = 0.05 level.

Among commercially insured individuals, there were two segments with a significant reduction in age-standardized VOC-related encounters (January 2011–March 2015; January 2020–April 2020) and two segments with increases in VOC-related encounters (March 2015–January 2020; April 2020–December 2022) (Fig. 1, Supplementary Material, eMethods 2). All age groups experienced increases in VOC-related encounters in the post-2020 years. Age-stratified analyses (Fig. 3) revealed that increases in VOC-related encounters, following the start of the COVID-19 pandemic, were more pronounced in younger age groups (MPC = 3.6% [95% CI: 2.7%, 4.9%] 1–12-year-old; 2.3% [95% CI: 0.2%, 13.8%] 13–17 year old) than older age groups (MPC = 0.6% [95% CI: 0.5%, 0.8%] 46–64 year old). Similar trends were observed in our secondary analyses (Supplementary Material, eMethods 6), illustrating post-COVID-19 increases in all-cause encounters were more pronounced in younger cohorts relative to older cohorts.

Among Medicaid beneficiaries, we similarly observed two segments with significant reductions in age-standardized VOC-related encounters (January 2011–September 2015; March 2016–December 2020), with two periods marked by significant increases in VOC-related encounters (September 2015–March 2016; December 2020–December 2022) (Fig. 1). Age-standardized monthly rates of VOC-related encounters increased by 0.6% from December 2020 to the end of 2022 (95% CI: 0.1%, 1.6%) (Fig. 1). Post-COVID-19 increases in VOC-related encounters were observed among younger Medicaid beneficiaries (MPC = 2.2% [95% CI: 1.6%, 3.1%] 1–12 year old; MPC = 1.0% [0.3%, 1.5%] 13–17 year old) but not older peers (MPC = −0.1% [−0.3%, −0.04%] for 46–64 year old) (Supplementary Material, eMethods 5).

As a supplemental analysis, we examined insurance- and sex-stratified age-standardized monthly rates of opioid prescribing and VOC-related acute care encounters (Supplemental Material, eMethods 3). In the commercial cohort, 53.7% of males (n = 4767) and 46.4% of females (n = 5880) had at least one VOC-related encounter; in the Medicaid cohort, these figures were 64.1% (n = 5937) and 45.9% (n = 6845), respectively. Temporal trajectories in opioid prescribing and VOC-related encounters were generally similar by sex (Supplementary Material, eMethods 3).

## Discussion

In this retrospective cohort study in the US, we observed a sustained decline in monthly outpatient opioid prescribing among individuals with SCD, decreasing from 19.1 to 13.5 per 100 individuals in the commercial cohort and from 32.5 to 13.6 in the Medicaid cohort between 2011 and 2022. Notably, these declines did not align consistently with increases in VOC-related acute care encounters during the pre-pandemic period. However, beginning in 2020, we observed a marked rise in VOC-related utilization: from 2020 to 2022, the average monthly percent change in VOC encounters was 0.6% in the Medicaid cohort and 1.8% in the commercial cohort, corresponding to an approximate annual increase of 7% and 21%, respectively. While these observational data cannot establish causality, the persistence and magnitude of these increases suggest they are unlikely to be due to random variation alone. These findings raise questions about whether changes in care delivery, access to pain management, or other disruptions during the COVID-19 pandemic may have contributed to rising acute care needs, particularly if declines in opioid prescribing were not matched by expanded access to alternative or timely pain management strategies.

To better understand these complex trends, it is helpful to examine how opioid prescribing and VOC-related encounters evolved over time in relation to major national policies. The 2016 CDC guidelines are often cited as a key factor influencing opioid prescribing and VOC admission trends in people with SCD. However, attributing these trends solely to one policy may oversimplify the multifactorial dynamics at play. In contrast to previous studies that estimated models anchored in 2016,^4^ our study employed joinpoint regression, a more flexible approach that identifies multiple shifts in trends without relying on a predefined change point. This allowed for a more nuanced analysis, particularly by including publicly insured individuals, a significant proportion of the SCD population. Notably, our models suggest that the downtrend in opioid prescribing observed among Medicaid beneficiaries likely preceded the 2016 guidelines, aligning with broader population-level data indicating that opioid prescribing peaked in 2012.^17^

Overlaying these policies and prescribing dynamics, our data also highlight the COVID-19 pandemic as a period of change, particularly for acute care utilization. The initial drop in VOC-related encounters, followed by a resurgence beginning mid-2020, aligns with previously reported trends of emergency department visits among people with SCD declining at the onset of the COVID-19 pandemic.^18^^,^^19^ Of concern, pediatric and young adult populations appeared to be significantly affected, with SCD-related readmission rates reaching their highest levels since 2017 in the years following the pandemic's onset.^6^ These findings underscore the broader impact of COVID-19 disruptions on health care access and pain management for individuals with SCD, especially those at younger ages.

Importantly, these pandemic-era trends did not occur uniformly across all individuals. In our analysis, insurance status emerged as an important axis of disparity in both opioid prescribing and VOC-related encounters. Many individuals living with SCD are publicly insured and covered by Medicaid^5^, ^6^, ^7^; therefore, understanding opioid utilization and SCD-related outcomes among both insurers is a strength of this analysis. Specifically, we found that Medicaid beneficiaries experienced higher overall rates of opioid prescribing and VOC-related encounters compared to the commercial cohort. Our data extend on preexisting literature suggesting that publicly insured individuals are more likely to have poorer SCD-related outcomes and higher health care utilization than commercially insured peers.^5^^,^^8^ Several factors may contribute to these differences. Individuals with frequent VOCs and chronic pain may have more difficulty securing or maintaining stable employment, which could limit access to employer-sponsored insurance.^20^ Another contributor to insurance-based disparities may be unstable insurance coverage among Medicaid beneficiaries, with studies illustrating that over 30% of young children with SCD enrolled in a multi-state database experienced interruptions or loss in coverage from 2015 to 2017.^21^ Further concerns exist about gaps in coverage during adolescence and young adulthood, a critical period associated with increased risk for SCD-related complications and higher healthcare utilization.^7^ Additionally, even individuals with stable insurance coverage may struggle to access specialty care. For instance, only 2–15% of Medicaid beneficiaries received care from a hematologist, compared to 39–47% among those with commercial insurance.^5^

Finally, we found age differences in SCD-related health care utilization across both insurance cohorts, such that adults, compared to children and adolescents, experienced higher rates of monthly opioid prescribing and VOC-related acute care encounters. Despite having lower rates of VOC-related encounters than adults, children and adolescents experienced the greatest increase in VOC-related acute care utilization following the COVID-19 pandemic's onset. The observed post-2020 rise in VOC-related encounters is likely multifactorial. Since these increases did not consistently correspond with declines in opioid prescribing, other factors, beyond medication access, may play a role. Pandemic-related disruptions in healthcare delivery, including reduced access to infusion centers and reliance on telemedicine, likely altered pain management approaches, particularly for children and adolescents. Heightened psychosocial stress during this period may have compounded these effects. Adolescents in the general population experienced marked increases in depression and anxiety during the COVID-19 pandemic, particularly those facing racial discrimination or limited access to care.^22^^,^^23^ Increased stress and anxiety, known triggers for VOCs,^24^^,^^25^ were particularly prevalent after 2020, a time period marked by heightened racism-based traumatic stress.^26^, ^27^, ^28^ Additionally, possible direct effects of SARS-CoV-2 infection on SCD pathophysiology may have worsened disease severity.^29^ Future research is needed to unpack how healthcare disruptions, psychosocial stressors, and systemic infections interact with VOC risk, especially among adolescents and young adults navigating both developmental transitions and structural inequities.^30^

### Limitations

Limitations are noted. First, our sample is limited to Medicaid or commercial enrollees, meaning that our findings cannot be generalized to uninsured people or those in other insurance plans (i.e., Medicare). Second, the MarketScan database records sex as a binary variable based on insurance claims data and does not capture gender identity or provide options for non-binary individuals. Third, we use a serial cross-sectional analysis of trends. While such a design, when combined with age-standardization, allows us to mitigate the risk of left truncation and death as a competing risk, it does not follow the same people at the individual level across months. Fourth, this is a study of outpatient opioid prescribing and does not assess whether individuals may receive opioids from inpatient pharmacies. Our ascertainment of VOCs via inpatient and emergency room visits may undercount crisis events that do not receive a VOC-related diagnosis or claim; for this reason, we conducted secondary analyses evaluating all-cause acute care encounters, whose findings were similar to those of the parent analyses (Supplementary Material, eMethods 6). We nonetheless are unable to capture home-managed VOCs. Fifth, MarketScan Multi-State Medicaid Database does not provide access to data on important structural determinants of health, including income, education, housing stability, neighborhood-level factors, or experiences of discrimination that may confound associations between race/ethnicity and health outcomes. There may be elements of structural racism that are not captured in our analyses (i.e., implicit racial bias, interpersonal racism) because they are unobservable in claims data. Future research that addresses multiple aspects of intersectionality (i.e., beyond insurance status and age) is needed to elucidate the systems of power that create barriers to healthcare utilization. Finally, the MarketScan data does not provide detailed information on opioid indications.

Despite these limitations, the present study is one of the only analyses to comprehensively evaluate opioid prescribing patterns and healthcare utilization across both public and private payers for individuals with SCD, using data spanning over a decade. As the US rolls out new cell-based gene therapies, our analysis provides much-needed estimates of baseline SCD-related healthcare utilization patterns in the pre-gene therapy era. While these therapies hold transformative potential, it remains unclear how they will affect chronic pain management and, consequently, healthcare utilization and opioid prescribing patterns. Additionally, disease-modifying therapies, which are outside the scope of the present manuscript, are critical treatments for long-term maintenance and prevention of VOCs. It is well-documented that disease-modifying therapies in SCD are underutilized, even with the addition of newer disease-modifying therapies in 2019.^11^ Additional research should evaluate how social determinants of health may impact access to both disease-modifying and curative therapies and influence SCD-related outcomes over time.

### Conclusions

In summary, our study provides a comprehensive analysis of trends in opioid prescribing and VOC-related acute care encounters among individuals with SCD from 2011 to 2022. We observed a sustained decline in opioid prescribing across both Medicaid beneficiaries and commercially insured individuals, a trend that began well before the 2016 CDC opioid prescribing guidelines. Notably, in the post-COVID-19 years, we identified increases in VOC-related acute care encounters, particularly among pediatric and young adult populations. These findings underscore the interplay of evolving healthcare policies, changing care delivery models, and the disruptive effects of the COVID-19 pandemic. While the decline in opioid prescribing was not consistently linked to increases in VOCs until after the pandemic, the underlying factors driving these trends remain unclear. By utilizing a flexible, data-driven approach to identify shifts in practice without relying on preconceived time points, this study offers new insights into the multifactorial dynamics affecting opioid use and acute care in a historically underserved population. The results highlight the need for more nuanced policy approaches that address both the challenges of pain management and the rising acute care needs of individuals with SCD, particularly in the context of age and insurance disparities. Future research should engage the SCD community to translate these findings into meaningful improvements in care delivery.

## Contributors

Conceptualization: KYX, RAG, CRJ, TVN, MRL, SAL; Research Question Development: KYX, RAG, CRJ, TVN, MRL, TP, LSM, EMN, SAL, TRB; Study Design: KYX, RAG, CRJ, TVN, MRL, TP, LSM, EMN, FD, JFS; Data Curation: KYX, JLB, DS; Data Management: KYX, JLB, DS; Formal Analysis of the MarketScan data: JLB, DS; Joinpoint Regressions with Tabulated Data: JS, RH; Visualization: JS, RH; Validation: JLB, FD, CAH, SAL; Resources: KYX, RAG, JFS; Supervision: KYX, RAG, JS, CRJ, TVN, JFS; Data Interpretation: All authors Initial Drafting: KYX, CRJ, TVN, LSM; Revision and approval of manuscript prior to initial submission: KYX, TVN, LSM, EMN, CAH, JLB, RH, FD, DS, JS, SAL, JFS, TRB, TP, MRL, RAG, CRJ; Revision of submission after peer review: KYX, RAG, TVN, CRJ, LG, JLB, LSM; Approval of Revised Manuscript: All authors; Access to Raw Data: KYX, JLB, DS; Access to Tabulated Data; All authors; KYX and CRJ had final responsibility for the decision to submit for publication.

## Data sharing statement

*Access and code access*: KYX, DS, and JLB have access to the proprietary data used in the analyses in the manuscript. Statistical code is available with a reasonable written request to the corresponding author via email. Data cleaning, linkage of inpatient, outpatient, and prescription data, and de-identification were overseen by Merative.

*Data availability*: The MarketScan data is available from Merative. Because the data is proprietary, the data can only be accessed via a data use agreement from Merative: https://www.merative.com/real-world-evidence.

## Declaration of interests

The authors declare no financial interests/personal relationships which may be considered as potential competing interests. EMN is an advisory board member/consultant for Novo Nordisk, Shield Therapeutics, Chiesi Pharmaceuticals. RAG reported receiving grants from the NIH and Arnold Ventures LLC during the conduct of the study, consulting for Janssen Pharmaceuticals, and receiving personal fees for grant reviews from the NIH outside the submitted work.
