# Supplementary material for: Ten-year trends in opioid prescribing and vaso-occlusive crises in sickle cell disease: a population-based national cohort study (2011–2022)
Source: Lancet Reg Health Am. 2025 Aug 21;50:101214. doi: 10.1016/j.lana.2025.101214 (PMC12396581; doi:10.1016/j.lana.2025.101214)
Supplement: Supplementary Materials [file mmc1.pdf]

Online-Only Supplement

eMethods 1: Administrative codes for key variables

eMethods 2: Detailed joinpoint regression output – Age-standardized monthly outpatient opioid prescribing and acute care encounters for VOCs, 2011-2022, stratified by insurance status

eMethods 3: Detailed joinpoint regression output – Age-standardized monthly outpatient opioid prescribing and acute care encounters for VOCs, 2011-2022, stratified by insurance status and sex

eMethods 4: Seasonal Decomposition- Age-standardized monthly outpatient opioid prescribing and acute care encounters for VOCs, 2011-2022, stratified by insurance status

eMethods 5: Detailed joinpoint regression output – Age- and insurance stratified monthly rates of outpatient opioid prescribing and acute care encounters for VOCs, 2011-2022

eMethods 6: Detailed joinpoint regression output – Age- and insurance stratified monthly rates of all-cause acute care encounters per month, 2011-2022

eMethods 1: Administrative codes for key variables

Coding of Opioid-Related Variables

<table>
<tr><th>Variable</th><th>Administrative Codes</th></tr>
<tr><td>Outpatient Prescription Opioids</td><td>
Outpatient prescriptions were queried using the generic name function in MarketScan (GENNME): 'FENTANYL' ‘CODEINE’ 'MORPHINE' 'METHADONE' 'OXYCODONE' 'OXYMORPHONE' 'HYDROCODONE' 'HYDROMORPHONE' 'PROPOXYPHENE' 'TRAMADOL', with data collected on continuous days’ supply, quantity of opioids prescribed, strength in the prescription, and morphine milligram equivalents (MMEs).
MME/day were calculated as the following product: Strength per unit x (Number of Units/ Quantity [days’ supply]) X MME conversion factor ; MME conversion factors can be obtained from CMS: https://www.hhs.gov/guidance/document/opioid-oral-morphine-milligram-equivalent-mme-conversion-factors-0
</td></tr>
<tr><td>Monthly Rate of Outpatient Opioid Prescriptions Filled</td><td>
Monthly rates of outpatient prescriptions filled were computed as the total number of opioid prescriptions filled per month divided by person-months in the month. Because absolute counts for opioid prescriptions and VOC-related admissions may be susceptible to time-varying exposure at the population level (see Hincapie-Castillo et al., 2023), the dependent variables were computed as percentages per 100 people and their estimated standard errors.
For our analyses, we estimated both age-stratified (eMethods 5-6) and age-standardized (eMethods 2-4) monthly rates.
Age-stratified monthly rates, which can also be referred to as “age-specific rates”, were calculated via stratification by age at time of prescription. In the context of SCD, where mortality rates vary by age, left truncation (see Debaun et al., 2019) arises when individuals must survive to a certain age to be eligible for study inclusion. This selection process can introduce bias if those who survive to older ages differ systematically from those who do not—often being healthier—which can lead to underestimation of disease burden or misrepresentation of outcome distributions.
Age standardization helps to partially mitigate this bias by re-weighting age-specific outcome estimates according to a standardized age distribution (e.g., using the age distribution in 2016 as the reference). This approach reduces confounding due to unequal age structures, which are a downstream consequence of differential survival. While age standardization does not directly model the left-truncated nature of the data, it adjusts the aggregated estimates to better reflect a common target population structure, thereby reducing distortions caused by overrepresentation of older, healthier survivors in the analytic sample. Nevertheless, we acknowledge that age standardization is a population-level adjustment and does not replace individual-level methods for addressing left truncation, such as survival models with delayed entry. However, it provides a conservative and interpretable approach that accounts for left truncation at the population level and helps ensure that our estimates are not unduly influenced by selective survival. To compute age-standardized rates, we used 2016 as the 'standard' age distribution to weigh the age-specific rates, as 2016 lies in the middle of the 2011-2022 study period. Age-standardized rates are calculated by multiplying the monthly age-specific rates by the corresponding weight and adding together to obtain a single age-adjusted rate per month.
Below is an example, using opioid prescriptions from January 2022 in the Commercial database.
<table>
<tr><td>Age Group</td><td>Commercial Database</td><td>Medicaid Database</td></tr>
<tr><td>1 to 12</td><td>0.142</td><td>0.279</td></tr>
<tr><td>13 to 17</td><td>0.089</td><td>0.137</td></tr>
<tr><td>18 to 27</td><td>0.193</td><td>0.257</td></tr>
<tr><td>28 to 45</td><td>0.330</td><td>0.230</td></tr>
<tr><td>46 to 64</td><td>0.243</td><td>0.090</td></tr>
</table>
<table>
<tr><td>Age Group</td><td>Number of opioid prescriptions initiated in January 2022</td><td>Number of SCD enrollees in January 2022</td><td>Age-specific rate per 100 SCD enrollees</td><td>Monthly weight</td><td>Age-adjusted rate per 100 SCD enrollees</td></tr>
<tr><td>1 to 12</td><td>44</td><td>462</td><td>9.524</td><td>0.142</td><td>2.38</td></tr>
<tr><td>13 to 17</td><td>57</td><td>368</td><td>15.489</td><td>0.089</td><td>10.87</td></tr>
<tr><td>18 to 27</td><td>127</td><td>795</td><td>15.975</td><td>0.193</td><td>16.60</td></tr>
<tr><td>28 to 45</td><td>200</td><td>1,455</td><td>13.746</td><td>0.330</td><td>14.02</td></tr>
<tr><td>46 to 64</td><td>73</td><td>1,197</td><td>6.099</td><td>0.243</td><td>9.52</td></tr>
</table>
January 2022 age-adjusted rate per 100 SCD enrollees:
</td></tr>
</table>

	(2.38*0.142) + (10.87*0.089) + (16.60*0.193) + (14.02*0.33) + (9.52*0.243) =11.44915<br><br>Reference:<br><br>DeBaun MR, Ghafuri DL, Rodeghier M, et al. Decreased median survival of adults with sickle cell disease after adjusting for left truncation bias: a pooled analysis. *Blood.* 2019;133(6):615-617.<br><br>Hincapie-Castillo JM, Goodin A. Using Joinpoint regression for drug utilization research: Tutorial and case study of prescription opioid use in the United States. *Pharmacoepidemiol Drug Saf.* 2023;32(5):509-516.

Coding of Sickle Cell Disease (SCD)

**Variable**	**Administrative Codes**
**Diagnosis of Sickle Cell Disease (SCD)**	We identified individuals with 1 inpatient (STDPLAC=20, 21, 23, 41, 42, 51) or 2 outpatient claims, at least 30 days apart, with a SCD diagnosis, at any point during insurance enrollment (between elig_start and elig_end in the diagram above) for any 1 of the following:<br><br>ICD-9/10 Codes: 282.41, 282.42, 282.6, D57.00, D57.01, D57.02, D57.211, D57.212, D57.219, D57.411, D57.412, D57.419, D57.811, D57.812, D57.819, D57.1, D57.20, D57.40, D57.80

Coding of Health Care Utilization

**Variable**	**Administrative Codes**
**Acute Care Encounters for Vaso-occlusive Crises (VOCs)**	We coded vaso-occlusive crises as acute care encounters, with any 1 of these ICD-9/10 Code for sickle cell disease with crisis (D57.0, D57.21, D57.419, D57.219, D57.81, D57.819, 282.62, 282.64, 282.69, 282.42). We limited encounters to emergency room encounters, encounters in urgent care, encounters in ambulance, and/or inpatient hospitalization [STDPLAC in (20, 21, 23, 41, 42, 51) OR days gt . or STDPROV in (1, 220, 428, 6)]
**All-Cause Acute Care Encounters**	We coded all-cause acute care encounters by querying claims for encounters that took place in emergency rooms, urgent care, ambulances, and/or hospitalization [STDPLAC in (20, 21, 23, 41, 42, 51) OR days gt . or STDPROV in (1, 220, 428, 6)]

For both all-cause and VOC-related acute care encounters, monthly rates were computed both via **standardization** (eMethods 2-4) and **stratification** by age at time of event (eMethods 5-6).

eMethods 2: Detailed joinpoint regression output – Age-standardized monthly outpatient opioid prescribing and acute care encounters for VOCs, 2011-2022, stratified by insurance status

This method uses **age-standardized rates a**s the input and identifies the month(s) when changes in trends are observed and subsequently calculates the monthly percentage change (MPC) in rates between trend-change points. The joinpoint method also estimates the average monthly percent change (AMPC) in the whole period studied. To compute the MPC, the following model is used: $\log(Yx)=b0+b1x$, where log (Yx) is the natural logarithm of the rate in month x. When there are no join points observed, the MPC is constant, thus equaling the AMPC. Otherwise, the time period is segmented by join points, with the AAMC estimated as a weighted average of the MPCs in each segment, using each segment's length as a weight.

Monthly Opioid Prescribing – Commercial

Months	Monthly Percentage Change (95%CI)	Jan 2011-Dec 2022 Monthly Average Percentage Change (95% CI)
Jan 2011-Apr 2012	-0.92% (-2.91%, -0.45%) *	-0.27% (-0.32%, -0.22%) *
Apr 2012-Jan 2021	-0.36% (-0.39%, -0.30%) *	
Jan 2021-Dec 2022	0.56% (0.18%, 1.25%) *	

Monthly Opioid Prescribing – Medicaid

Months	Monthly Percentage Change (95%CI)	Jan 2011-Dec 2022 Monthly Average Percentage Change (95% CI)
Jan 2011-Apr 2012	-1.66% (-2.93%, -1.08%) *	-0.54% (-0.64%, -0.45%) *
Apr 2012-Jan 2016	-0.48% (-0.57%, -0.29%) *	
Jan 2016-June 2022	-1.00% (-1.13%, -0.91%) *	
June 2022-Dec 2022	7.17% (3.11%, 15.40%) *	

Monthly VOC-related Acute Care Encounters – Commercial

Months	Monthly Percentage Change (95%CI)	Jan 2011-Dec 2022 Monthly Average Percentage Change (95% CI)
Jan 2011-Mar 2015	-0.46% (-0.64%, -0.33%) *	0.14% (0.10%, 0.19%) *
Mar 2015-Jan 2020	0.21% (0.10%, 0.41%) *	
Jan 2020-Apr 2020	-7.96% (-9.88%, -1.15%) *	
Apr 2020-Dec 2022	1.76% (1.48%, 2.15%) *	

Monthly VOC-related Acute Care Encounters – Medicaid

Months	Monthly Percentage Change (95%CI)	Jan 2011-Dec 2022 Monthly Average Percentage Change (95% CI)
Jan 2011-Sept 2015	-0.16% (-0.38%, -0.04%) *	-0.07% (-0.12%, -0.01%) *
Sept 2015-Mar 2016	1.88% (0.02%, 5.03%) *	
Mar 2016-Jan 2020	-0.45% (-0.69%, -0.35%) *	
Jan 2020-Dec 2022	0.60% (0.11%, 1.58%) *	

*: Indicate MPC (monthly percent change) and AMPC (average monthly percentage change) are significantly different from zero at the alpha = 0.05 level

eMethods 3: Detailed joinpoint regression output – Age-standardized monthly outpatient opioid prescribing and acute care encounters for VOCs, 2011-2022, stratified by insurance status and sex

We further stratified the analyses in eMethods 2 by sex. Among commercially insured individuals, 64.7% of females (n=8,195) and 60.8% of males (n=5,401) had at least one outpatient opioid prescription during the study period, with a mean number of prescriptions per 100 individuals per month of 1.2 (SD=0.6) and 1.3 (SD=0.6), respectively. In the Medicaid cohort, 53.6% of females (n=8,007) and 58.4% of males (n=5,409) had at least one opioid prescription, with mean monthly prescribing rates of 1.4 prescriptions per 100 individuals (SD=0.8) for both groups. Regarding VOC-related acute care encounters, 46.4% of females (n=5,880) and 53.7% of males (n=4,767) in the commercial cohort had at least one VOC-related encounter, with mean monthly rates of 1.4 (SD=0.9) and 1.3 (SD=0.7) per 100 individuals, respectively. In the Medicaid cohort, 45.9% of females (n=6,845) and 64.1% of males (n=5,937) had at least one VOC-related encounter, with both groups exhibiting a mean of 1.6 encounters per month per 100 individuals (SD=1.1 for males and SD=1.2 for females).

As in eMethods 2, we used **age-standardized** monthly rates as inputs, identifying inflection points in trends and calculating the monthly percentage change (MPC) between those time segments using joinpoint regression models. In all models, an asterisk (*) indicates that the MPC or AMPC (average MPC) is significantly different from zero at α=0.05. Among commercially insured individuals, prescribing declined from January 2011 through December 2022 at similar rates for males (MPC = –0.27%, 95% CI: –0.32%, –0.21%) and females (MPC = –0.27%, 95% CI: –0.31%, –0.23%). Among Medicaid beneficiaries, females showed greater reductions in prescribing (MPC = –0.61%, 95% CI: –0.73%, –0.51%) compared to males (MPC = –0.35%, 95% CI: –0.44%, –0.26%). For both insurance types, these downward trends were concentrated in the pre-2020 period and either attenuated or reversed thereafter.

Trends in VOC-related acute care encounters demonstrated similar patterns across sexes within insurance types. Among commercially insured individuals, both males and females experienced significant increases in VOC encounters post-2020, with males showing an MPC of 1.91% (95% CI: 1.55%, 2.44%) and females an MPC of 1.73% (95% CI: 1.40%, 2.22%) in the years following the COVID-19 public health emergency.. In the Medicaid cohort, declining VOC encounter trends observed since 2016 were not observed after 2020; post-2020 increases in monthly VOCs among females reached statistical significance (MPC = 0.60%, 95% CI: 0.13%, 1.54%), while the corresponding increase among males (MPC = 0.38%, 95% CI: –0.01%, 1.29%) did not.

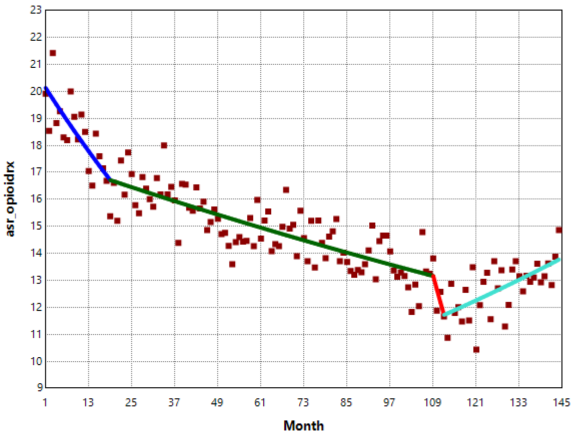

Monthly Opioid Prescribing – Commercial, Male		
Months	Monthly Percentage Change (95%CI)	Jan 2011-Dec 2022 Monthly Average Percentage Change (95% CI)
Jan 2011-Jul 2012	-1.03% (-1.97%, -0.50%) *	-0.27% (-0.32%, -0.21%) *
Jul 2012-Jan 2020	-0.27% (-0.33%, 1.25%)	
Jan 2020-Apr 2020	-3.80% (-5.24%, -0.25%) *	
Apr 2020-Dec 2022	0.51% (0.18%, 1.03%) *	

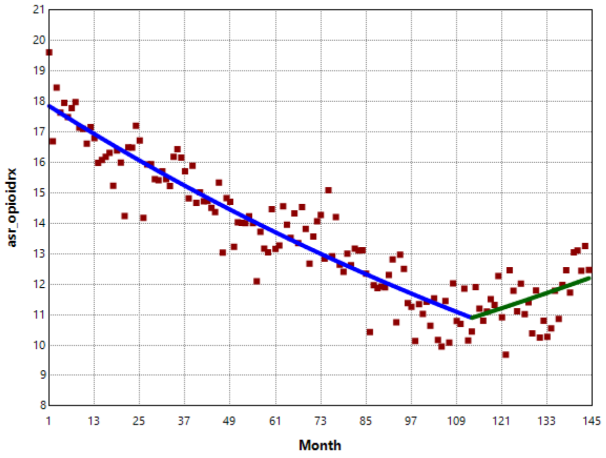

Monthly Opioid Prescribing – Commercial, Female		
Months	Monthly Percentage Change (95%CI)	Jan 2011-Dec 2022 Monthly Average Percentage Change (95% CI)
Jan 2011-May 2020	-0.44% (-0.48%, -0.41%) *	-0.27% (-0.31%, -0.23%) *
May 2020-Dec 2022	0.37% (0.09%, 0.80%) *	

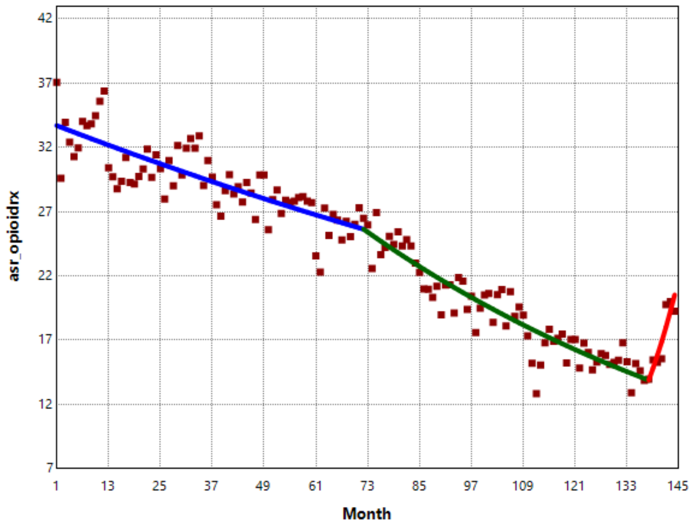

Monthly Opioid Prescribing – Medicaid, Male		
Months	Monthly Percentage Change (95%CI)	Jan 2011-Dec 2022 Monthly Average Percentage Change (95% CI)
Jan 2011-Dec 2016	-0.38% (-0.45%, -0.31%) *	-0.35% (-0.44%, -0.26%) *
Dec 2016-Jun 2022	-0.92% (-1.06%, -0.83%) *	
Jun 2022-Dec 2022	6.72% (2.73%, 14.85%) *	

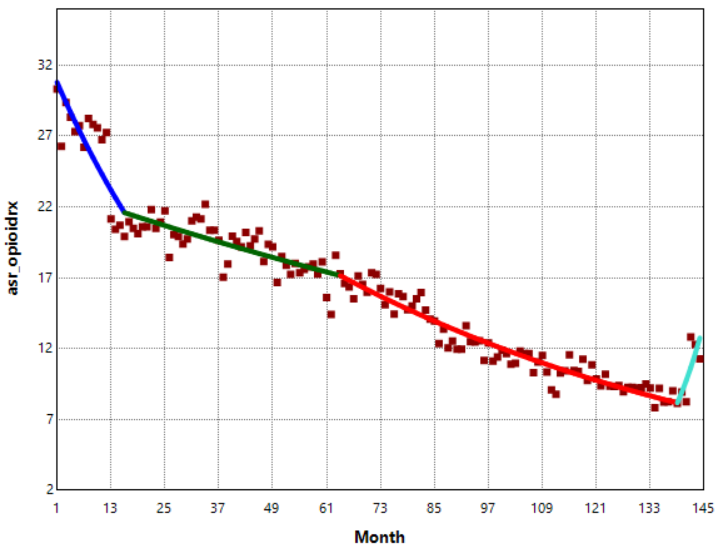

Monthly Opioid Prescribing – Medicaid, Female		
Months	Monthly Percentage Change (95%CI)	Jan 2011-Dec 2022 Monthly Average Percentage Change (95% CI)
Jan 2011-Apr 2012	-2.34% (-3.22%, -1.77%) *	-0.61% (-0.73%, -0.51%) *
Apr 2012-Apr 2016	-0.48% (-0.59%, -0.28%) *	
Apr 2016-Jul 2022	-0.98% (-1.11%, -0.91%) *	
Jul 2022-Dec 2022	9.32% (3.46%, 21.79%) *	

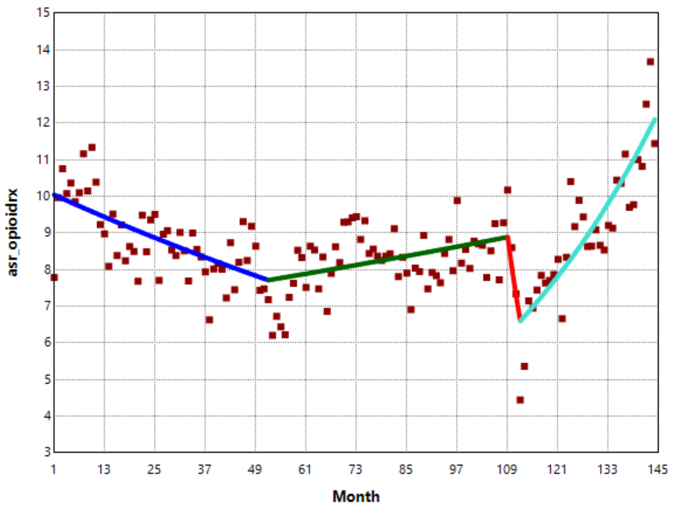

Monthly VOC-related Acute Care Encounters – Commercial, Male		
Months	Monthly Percentage Change (95%CI)	Jan 2011-Dec 2022 Monthly Average Percentage Change (95% CI)
Jan 2011-Apr 2015	-0.52% (-0.75%, -0.34%) *	0.13% (0.07%, 0.19%) *
Apr 2015-Jan 2020	0.25% (0.11%, 0.61%) *	
Jan 2020-Apr 2020	-9.45% (-11.95%, -0.97%) *	
Apr 2020-Dec 2022	1.91% (1.55%, 2.44%) *	

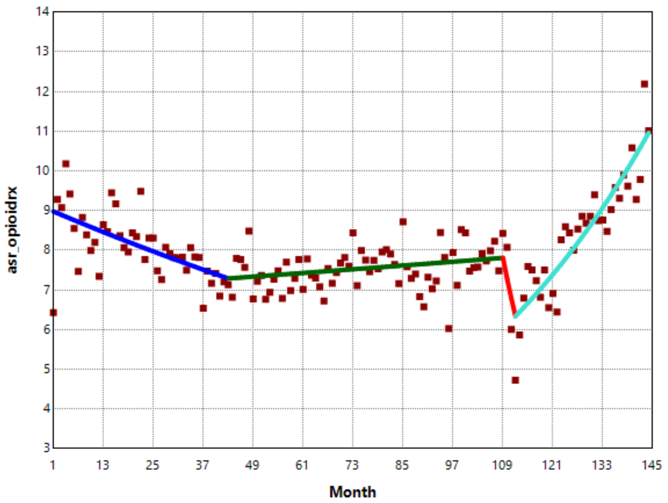

Monthly VOC-related Acute Care Encounters – Commercial, Female		
Months	Monthly Percentage Change (95%CI)	Jan 2011-Dec 2022 Monthly Average Percentage Change (95% CI)
Jan 2011-Jul 2014	-0.50% (-0.82%, -0.28%) *	0.14% (0.08%, 0.20%) *
Jul 2014-Jan 2020	0.10% (-0.01%, 0.77%)	
Jan 2020-Apr 2020	-6.72% (-8.75%, -0.08%) *	
Apr 2020-Dec 2022	1.73% (1.40%, 2.22%) *	

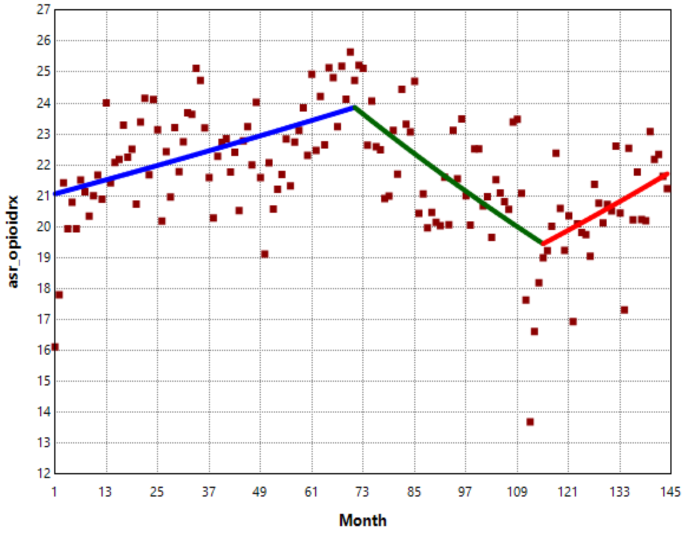

Monthly VOC-related Acute Care Encounters – Medicaid, Male		
Months	Monthly Percentage Change (95%CI)	Jan 2011-Dec 2022 Monthly Average Percentage Change (95% CI)
Jan 2011-Nov 2016	0.18% (0.10%, 0.30%) *	0.02% (-0.03%, 0.08%)
Nov 2016-Jul 2020	-0.46% (-2.55%, -0.31%) *	
Jul 2020-Dec 2022	0.38% (-0.01%, 1.29%)	

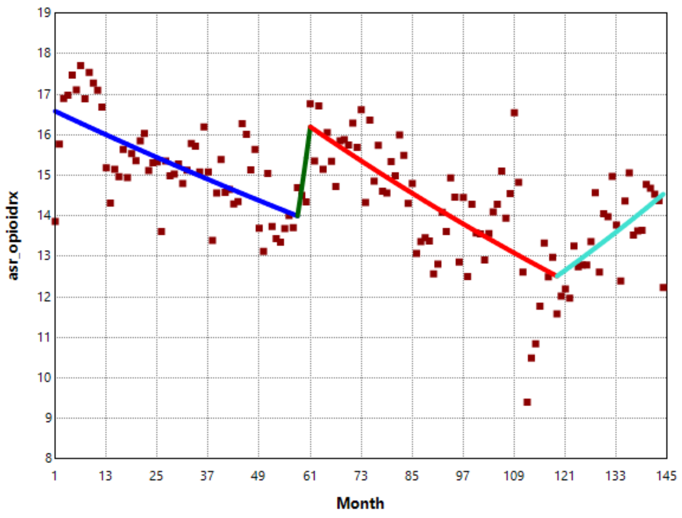

Monthly VOC-related Acute Care Encounters – Medicaid, Female		
Months	Monthly Percentage Change (95%CI)	Jan 2011-Dec 2022 Monthly Average Percentage Change (95% CI)
Jan 2011-Oct 2015	-0.30% (-0.44%, -0.20%) *	-0.09% (-0.15%, -0.04%) *
Oct 2015-Jan 2016	4.99% (0.44%, 6.43%) *	
Jan 2016-Nov 2020	-0.44% (-0.65%, -0.35%) *	
Nov 2020-Dec 2022	0.60% (0.13%, 1.54%) *	

eMethods 4: Seasonal Decomposition – Age-standardized outpatient opioid prescribing and acute care encounters for VOCs, 2011-2022, stratified by insurance status

Seasonal Decomposition: Monthly Opioid Prescribing – Commercial			Parent Models: Monthly Opioid Prescribing – Commercial		
**Month**	**MPC (95%CI)**	**2011.1-2022.12 AMPC**	**Month**	**MPC (95%CI)**	**2011.1-2022.12 AMPC**
Jan 2011-Mar 2012	-0.99%* (-2.14%, -0.59%)	-0.28*<br>(-0.32, -0.24)	Jan 2011-Apr 2012	-0.92%* (-2.91%, -0.45%)	-0.27%*<br>(-0.32%, -0.22%)
Mar 2012-Nov 2020	-0.36%* (-0.39%, -0.33%)		Apr 2012-Jan 2021	-0.36%* (-0.39%, -0.30%)	
Nov 2020-Dec 2022	0.46%* (0.19%, 0.86%)		Jan 2021-Dec 2022	0.56%* (0.18%, 1.25%)	

Seasonal Decomposition: Monthly Opioid Prescribing – Medicaid			Parent Models: Monthly Opioid Prescribing – Medicaid		
**Month**	**MPC (95%CI)**	**2011.1-2022.12 AMPC**	**Month**	**MPC (95%CI)**	**2011.1-2022.12 AMPC**
Jan 2011-Apr 2012	-1.75%* (-2.53%, -1.30%)	-0.58%*<br>(-0.67%, -0.50%)	Jan 2011-Apr 2012	-1.66%* (-2.93%, -1.08%)	-0.54%*<br>(-0.64%, -0.45%)
Apr 2012-May 2016	-0.45%* (-0.53%, -0.31%)		Apr 2012-Jan 2016	-0.48%* (-0.57%, -0.29%)	
May 2016-Jul 2022	-0.95%* (-1.03%, -0.89%)		Jan 2016-June 2022	-1.00%* (-1.13%, -0.91%)	
Jul 2022-Dec 2022	7.40%* (2.90%, 16.96%)		June 2022-Dec 2022	7.17%* (3.11%, 15.40%)	

Seasonal Decomposition: Monthly VOC-related Acute Care Encounters – Commercial			Parent Models: Monthly VOC-related Acute Care Encounters – Commercial		
**Months**	**MPC (95%CI)**	**2011.1-2022.12 AMPC**	**Month**	**MPC (95%CI)**	**2011.1-2022.12 AMPC**
Jan 2011-Aug 2014	-0.55%* (-0.77%, -0.38%)	0.13%*<br>(0.08%, 0.17%)	Jan 2011-Mar 2015	-0.46%* (-0.64%, -0.33%)	0.14%*<br>(0.10%, 0.19%)
Aug 2014-Jan 2020	0.15%* (0.07%, 0.31%)		Mar 2015-Jan 2020	0.21%* (0.10%, 0.41%)	
Jan 2020-Apr 2020	-7.38%* (-9.22%, -0.94%)		Jan 2020-Apr 2020	-7.96%* (-9.88%, -1.15%)	
Apr 2020-Dec 2022	1.72%* (1.44%, 2.11%)		Apr 2020-Dec 2022	1.76%* (1.48%, 2.15%)	

Seasonal Decomposition: Monthly VOC-related Acute Care Encounters – Medicaid			Parent Models: Monthly VOC-related Acute Care Encounters – Medicaid		
**Month**	**MPC (95%CI)**	**2011.1-2022.12 AMPC**	**Month**	**MPC (95%CI)**	**2011.1-2022.12 AMPC**
Jan 2011-Nov 2015	-0.17%* (-0.28%, -0.09%)	-0.07%*<br>(-0.12%, -0.03%)	Jan 2011-Sept 2015	-0.16%* (-0.38%, -0.04%)	-0.07%*<br>(-0.12%, -0.01%)
Nov 2015-Feb 2016	4.20%* (0.46%, 5.31%)		Sept 2015-Mar 2016	1.88%* (0.02%, 5.03%)	
Feb 2016-Nov 2020	-0.46%* (-0.60%, -0.39%)		Mar 2016-Jan 2020	-0.45%* (-0.69%, -0.35%)	
Nov 2020-Dec 2022	0.54%* (0.20%, 1.19%)		Jan 2020-Dec 2022	0.60%* (0.11%, 1.58%)	

*: Indicate MPC (monthly percent change) and AMPC (average monthly percentage change) are significantly different from zero at the alpha = 0.05 level

Reference for more details on method: Cleveland RB, Cleveland WS, McRae JE, Terpenning I. 1990. Stl: A seasonal-trend decomposition procedure based on loess. *J Official Statistics*, 6(1):3–73.

eMethods 5: Detailed joinpoint regression output – Age- and insurance stratified rates of outpatient opioid prescribing and VOC-related acute care encounters, 2011-2022

Monthly Opioid Prescribing – Commercial			
Cohort	Months	Monthly Percentage Change (95%CI)	Jan 2011-Dec 2022 Monthly Average Percentage Change (95%CI)
1 to 12 years	Jan 2011-May 2017	0.43% (0.29%, 0.62%) *	-0.09% (-0.19%, -0.01%) *
	May 2017-Dec 2022	-0.68% (-1.01%, -0.46%) *	
13 to 17 years	Jan 2011-Dec 2022	-0.17% (-0.25%, -0.11%) *	-0.17% (-0.25%, -0.11%) *
18 to 27 years	Jan 2011-Apr 2012	-1.81% (-3.32%, -1.09%) *	-0.19% (-0.27%, -0.12%) *
	Apr 2012-Feb 2021	-0.28% (-0.34%, -0.23%) *	
	Feb 2021-Dec 2022	1.37% (0.77%, 2.36%) *	
28 to 45 years	Jan 2011-Apr 2020	-0.46% (-0.50%, -0.42%) *	-0.29% (-0.34%, -0.25%) *
	Apr 2020-Dec 2022	0.29% (0.01%, 0.77%) *	
46 to 64 years	Jan 2011-Dec 2019	-0.46% (-0.55%, -0.42%) *	-0.36% (-0.41%, -0.29%) *
	Dec 2019-Dec 2022	-0.04% (-0.32%, 1.11%)	

Monthly Opioid Prescribing – Medicaid			
Cohort	Months	Monthly Percentage Change (95%CI)	Jan 2011-Dec 2022 Monthly Average Percentage Change (95%CI)
1 to 12 years	Jan 2011-Sept 2016	0.45% (0.34%, 0.61%) *	-0.04% (-0.30%, 0.15%)
	Sept 2016-Sept 2019	-0.55% (-1.15%, -0.01%) *	
	Sept 2019-July 2022	-2.08% (-10.46%, -1.51%) *	
	July 2022-Dec 2022	11.65% (-0.38%, 37.55%)	
13 to 17 years	Jan 2011-Aug 2022	-0.25% (-0.33%, -0.20%) *	-0.001% (-0.20%, 0.15%)
	Aug 2022-Dec 2022	8.99% (-0.17%, 29.02%)	
18 to 27 years	Jan 2011-Sept 2017	-0.58% (-0.64%, -0.51%) *	-0.68% (-0.75%, -0.61%) *
	Sept 2017-Apr 2020	-1.47% (-6.19%, -1.15%) *	
	Apr 2020-Dec 2022	-0.18% (-0.66%, 0.80%)	
28 to 45 years	Jan 2011-Mar 2013	-1.93% (-2.31%, -1.69%) *	-0.73% (-0.82%, -0.65%) *
	Mar 2013-July 2013	2.91% (-0.36%, 5.69%)	
	July 2013-Apr 2022	-1.01% (-1.07%, -0.97%) *	
	Apr 2022-Dec 2022	5.18% (2.52%, 10.23%) *	
46 to 64 years	Jan 2011-Apr 2012	-3.23% (-4.15%, -2.54%) *	-0.49% (-0.60%, -0.39%) *
	Apr 2012-Oct 2013	0.97% (0.43%, 2.12%) *	
	Oct 2013-July 2022	-0.93% (-0.99%, -0.89%) *	
	July 2022-Dec 2022	12.74% (6.98%, 22.27%) *	

Monthly VOC-related Acute Care Encounters – Commercial			
Cohort	Months	Monthly Percentage Change (95%CI)	Jan 2011-Dec 2022 Monthly Average Percentage Change (95%CI)
1 to 12 years	Jan 2011-Jan 2020	0.02% (-0.08%, 0.11%)	0.22% (0.10%, 0.34%) *
	Jan 2020-Apr 2020	-24.58% (-29.23%, -5.48%) *	

	Apr 2020-Dec 2022	3.62% (2.72%, 4.92%) *	
13 to 17 years	Jan 2011-July 2021	-0.10% (-0.26%, -0.02%) *	0.18% (0.004%, 0.37%) *
	July 2021-Dec 2022	2.30% (0.24%, 13.84%) *	
18 to 27 years	Jan 2011-Feb 2014	-0.72% (-1.49%, 0.74%)	0.06% (-0.04%, 0.16%)
	Feb 2014-Jan 2020	-0.05% (-3.47%, 4.17%)	
	Jan 2020-Apr 2020	-5.84% (-8.46%, 3.97%)	
	Apr 2020-Dec 2022	1.78% (0.40%, 2.67%) *	
28 to 45 years	Jan 2011-Mar 2011	23.06% (-0.48%, 39.16%)	0.45% (0.22%, 0.64%) *
	Mar 2011-Jan 2014	-0.89% (-1.65%, 0.15%)	
	Jan 2014-Dec 2021	0.22% (0.10%, 0.31%) *	
	Dec 2021-Dec 2022	2.77% (1.15%, 7.39%) *	
46 to 64 years	Jan 2011-Dec 2013	0.31% (-0.10%, 1.02%)	0.25% (0.18%, 0.33%) *
	Dec 2013-May 2015	-1.94% (-10.88%, -0.79%) *	
	May 2015-Dec 2022	0.64% (0.53%, 0.76%) *	

**Monthly VOC-related Acute Care Encounters - Medicaid**			
Cohort	Months	Monthly Percentage Change (95%CI)	Jan 2011-Dec 2022 Monthly Average Percentage Change (95%CI)
1 to 12 years	Jan 2011-Jan 2020	0.19% (0.11%, 0.27%) *	0.27% (0.18%, 0.37%) *
	Jan 2020-Apr 2020	-15.48% (-18.94%, -2.71%) *	
	Apr 2020-Dec 2022	2.19% (1.56%, 3.13%) *	
13 to 17 years	Jan 2011-Feb 2020	0.47% (0.40%, 0.56%) *	0.47% (0.39%, 0.54%) *
	Feb 2020-May 2020	-22.87% (-28.45%, -10.43%) *	
	May 2020-Aug 2020	25.38% (8.58%, 35.97%) *	
	Aug 2020-Dec 2022	0.95% (0.30%, 1.45%) *	
18 to 27 years	Jan 2011-May 2011	7.41% (0.48%, 21.44%) *	-0.02% (-0.15%, 0.12%)
	May 2011-Nov 2017	-0.08% (-0.18%, 0.004%)	
	Nov 2017-Feb 2018	-4.91% (-6.25%, -0.45%) *	
	Feb 2018-Dec 2022	-0.17% (-0.30%, 0.07%)	
28 to 45 years	Jan 2011-Apr 2015	-0.39% (-0.56%, -0.27%) *	-0.21% (-0.25%,-0.17%) *
	Apr 2015-Aug 2016	0.82% (0.23%, 5.36%) *	
	Aug 2016-May 2019	-0.74% (-1.64%, -0.53%) *	
	May 2019-Dec 2022	0.04% (-0.12%, 0.28%)	
46 to 64 years	Jan 2011-Dec 2015	-0.61% (-0.90%, -0.41%) *	-0.15% (-0.23%, -0.09%) *
	Dec 2015-May 2016	5.25% (0.72%, 11.57%) *	
	May 2016-Dec 2022	-0.14% (-0.30%, -0.04%) *	

*: Indicate MPC (monthly percent change) and AMPC (average monthly percentage change) are significantly different from zero at the alpha = 0.05 level

eMethods 6: Detailed joinpoint regression output – Age- and insurance stratified rates of all-cause acute care encounters per month, 2011-2022

Monthly All Cause Acute Care Encounters – Commercial			
**Cohort**	**Months**	**Monthly Percentage Change (95%CI)**	**Jan 2011-Dec 2022 Monthly Average Percentage Change (95%CI)**
1 to 12 years	Jan 2011-Mar 2011	48.23% (22.51%, 65.52%) *	0.62% (0.44%, 0.79%) *
	Mar 2011-Jan 2020	-0.05%(-0.11%, 0.01%)	
	Jan 2020-Apr 2020	-18.75% (-21.59%, -5.86%) *	
	Apr 2020-Dec 2022	2.42% (1.90%, 3.09%) *	
13 to 17 years	Jan 2011-Mar 2011	37.10% (11.09%, 55.44%) *	0.69% (0.49%, 0.88%) *
	Mar 2011-Jan 2020	0.04% (-0.02%, 0.12%)	
	Jan 2020-Apr 2020	-9.69% (-12.15%, -0.69%) *	
	Apr 2020-Dec 2022	1.94% (1.52%, 2.61%) *	
18 to 27 years	Jan 2011-Oct 2020	-0.14% (-0.20%, 0.25%)	0.03% (-0.06%, 0.13%)
	Oct 2020-Apr 2021	3.54% (-2.34%, 9.02%)	
	Apr 2021-Dec 2022	-0.02% (-2.45%, 0.86%)	
28 to 45 years	Jan 2011-Mar 2011	28.26% (9.62%, 44.07%) *	0.61% (0.45%, 0.77%) *
	Mar 2011-Jan 2020	0.09% (0.02%, 0.18%) *	
	Jan 2020-Apr 2020	-4.41% (-6.22%, 2.70%)	
	Apr 2020-Dec 2022	1.29% (-0.28%, 1.94%)	
46 to 64 years	Jan 2011-June 2011	11.92% (4.81%, 29.16%) *	0.61% (0.46%, 0.82%) *
	June 2011-Apr 2015	-0.07% (-1.77%, 0.16%)	
	Apr 2015-Dec 2022	0.36% (0.28%, 0.51%) *	

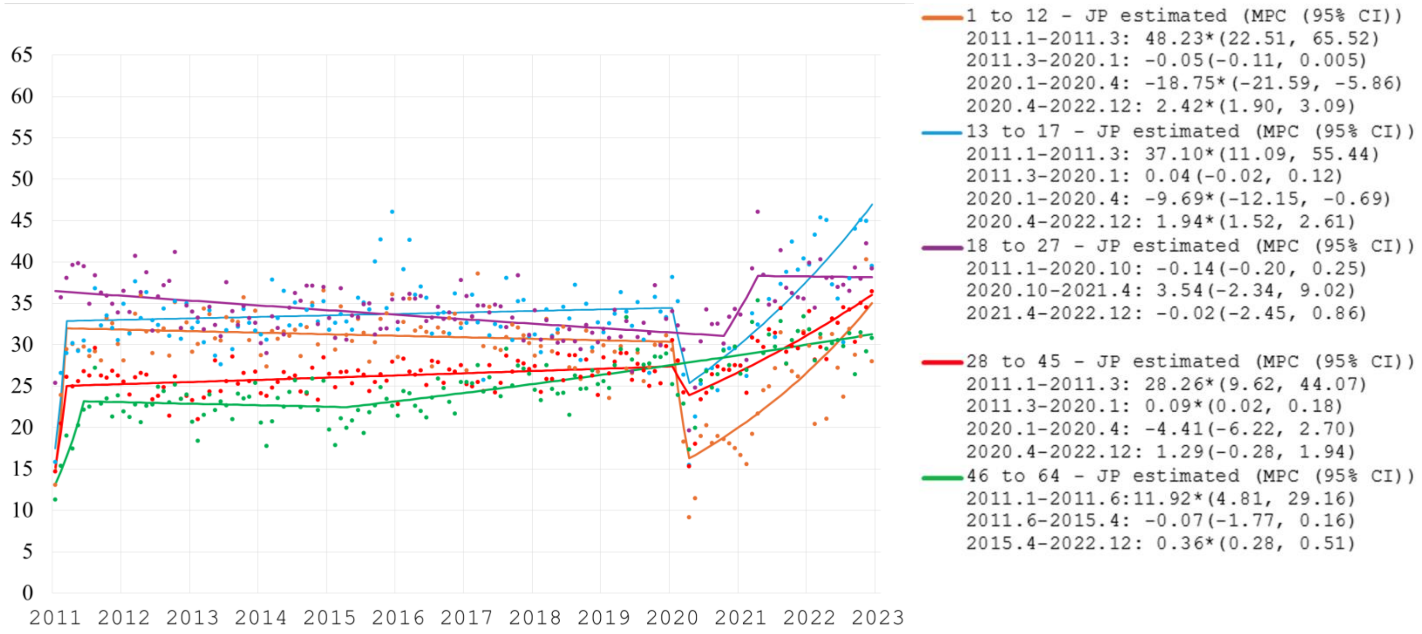

*: Indicate MPC (monthly percent change) and AMPC (average monthly percentage change) are significantly different from zero at the alpha = 0.05 level

Monthly All Cause Acute Care Encounters - Medicaid			
**Cohort**	**Month**	**Monthly Percentage Change (95%CI)**	**Jan 2011-Dec 2022 Monthly Average Percentage Change (95%CI)**
1 to 12 years	Jan 2011-Nov 2012	1.41% (0.91%, 2.23%) *	0.26% (0.18%, 0.35%) *
	Nov 2012-Jan 2020	-0.01% (-0.09%, 0.06%)	
	Jan 2020-Apr 2020	-11.57% (-13.95%, -2.44%) *	
	Apr 2020-Dec 2022	1.39% (0.94%, 2.05%) *	
13 to 17 years	Jan 2011-Mar 2011	43.27% (10.32%, 66.72%) *	0.75% (0.52%, 0.97%) *
	Mar 2011-Jan 2020	0.21% (0.15%, 0.27%) *	
	Jan 2020-Apr 2020	-8.65% (-10.58%, -1.16%) *	
	Apr 2020-Dec 2022	1.28% (0.92%, 1.80%) *	
18 to 27 years	Jan 2011-May 2013	0.76% (0.50%, 1.26%) *	0.001% (-0.04%, 0.06%)
	May 2013-Oct 2017	-0.06% (-0.16%, 0.05%)	
	Oct 2017-Mar 2018	-3.18% (-6.14%, -0.79%) *	
	Mar 2018-Dec 2022	-0.04%(-0.15%, 0.14%)	
28 to 45 years	Jan 2011-Jan 2016	0.38% (0.29%, 0.50%) *	0.06% (0.02%, 0.10%) *
	Jan 2016-May 2020	-0.46% (-0.65%, -0.37%) *	
	May 2020-Dec 2022	0.30% (0.05%, 0.73%) *	
46 to 64 years	Jan 2011-July 2015	0.92% (0.76%, 1.21%) *	0.41% (0.36%, 0.49%) *
	July 2015-Jan 2020	0.08% (-0.07%, 0.34%)	
	Jan 2020-Apr 2020	-4.97% (-6.60%, -0.04%) *	
	Apr 2020-Dec 2022	0.65% (0.31%, 1.27%) *	

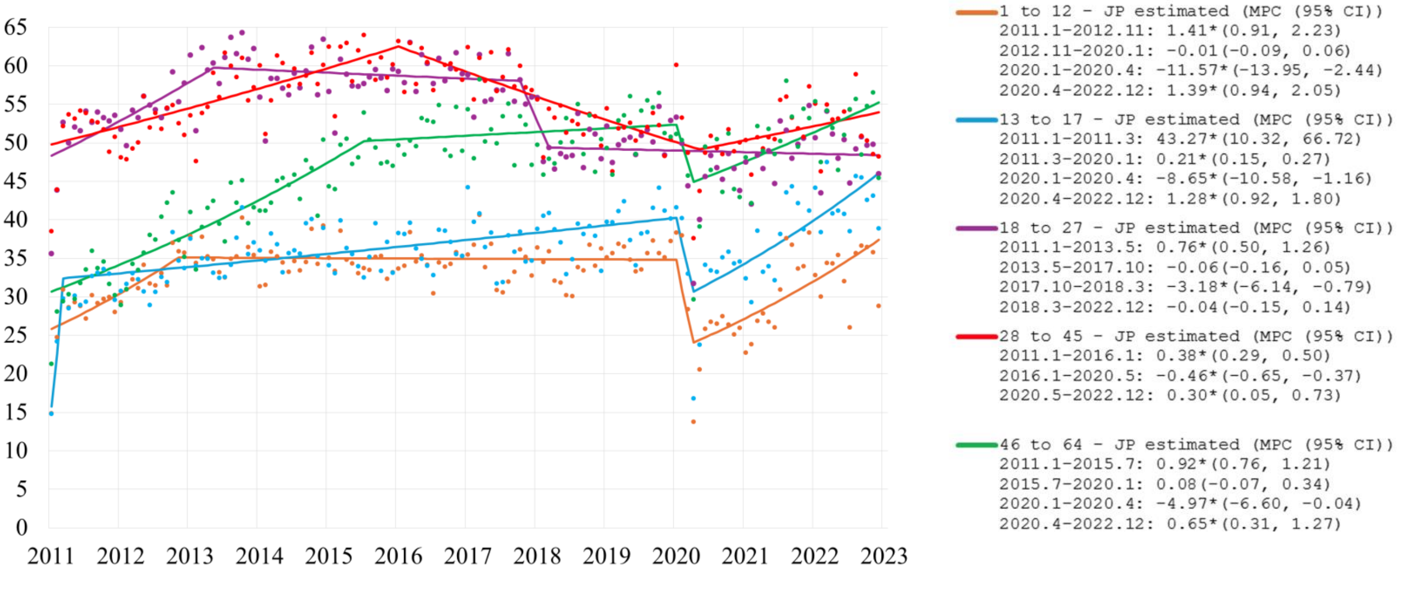

*: Indicate MPC (monthly percent change) and AMPC (average monthly percentage change) are significantly different from zero at the alpha = 0.05 level
